# β-Lactoglobulin Enhances Clay and Activated Carbon Binding and Protection Properties for Cadmium and Lead

**DOI:** 10.1021/acs.iecr.4c01774

**Published:** 2024-09-06

**Authors:** Kendall Lilly, Meichen Wang, Asuka A. Orr, Sarah E. Bondos, Timothy D. Phillips, Phanourios Tamamis

**Affiliations:** † Department of Materials Science and Engineering, College of Engineering, Texas A&M University, College Station, Texas 77843, United States; ‡ Artie McFerrin Department of Chemical Engineering, College of Engineering, Texas A&M University, College Station, Texas 77843, United States; § Department of Veterinary Physiology and Pharmacology, College of Veterinary Medicine and Biomedical Sciences, Texas A&M University, College Station, Texas 77843, United States; ∥ Interdisciplinary Faculty of Toxicology, College of Veterinary Medicine and Biomedical Sciences, Texas A&M University, College Station, Texas 77843, United States; ⊥ Department of Medical Physiology Texas A&M Health Science Center, Texas A&M University, College Station, Texas 77843, United States; # Department of Environmental Health Sciences, University of Massachusetts Amherst, Amherst, Massachusetts 01003, United States

## Abstract

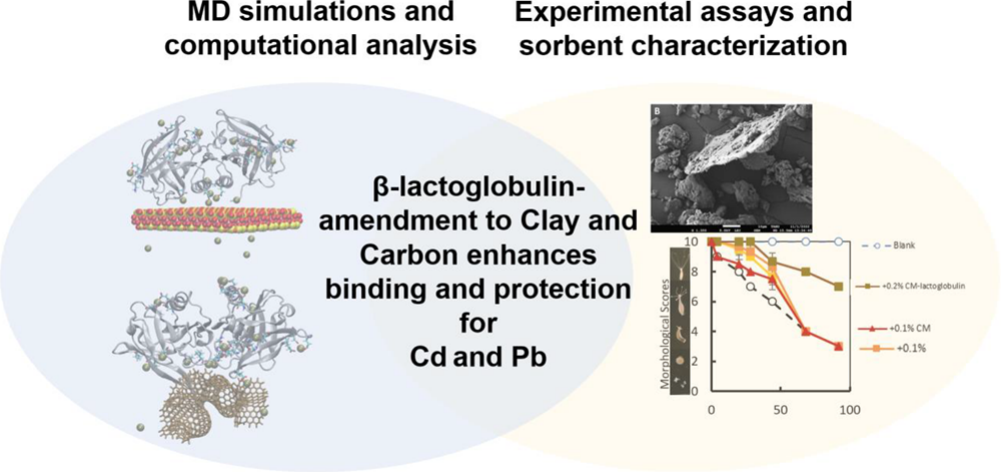

The removal of heavy metals from wastewater remains a challenge due to the limitations of current remediation methods. This study aims to develop multicomponent composites as inexpensive and environmentally friendly sorbents with enhanced capture of cadmium (Cd) and lead (Pb). The composites are based on calcium montmorillonite (CM) and activated carbon (AC) because of their proven effectiveness as sorbents for diverse toxins in environmental settings. In this study, we used a combination of computational and experimental methods to delineate that β-lactoglobulin enhances CM and AC binding and protection properties for Cd and Pb. Modeling and molecular dynamics simulations investigated the formation of material systems formed by CM and AC in complex with β-lactoglobulin and predicted their capacity to bind heavy metal ions at neutral pH conditions. Our simulations suggest that the enhanced binding properties of the material systems are attributed to the presence of several binding pockets formed by β-lactoglobulin for the two heavy metal ions. At neutral pH conditions, divalent Cd and Pb shared comparable binding propensities in all material systems, with the former being consistently higher than the latter. To validate the interactions depicted in simulations, two ecotoxicological models (*L. minor* and *H. vulgaris*) were exposed to Cd, Pb, and a mixture of the two. The inclusion of CM-lactoglobulin (β-lactoglobulin amended CM) and AC-lactoglobulin (β-lactoglobulin amended AC) at 0.05–0.2% efficiently and dose-dependently reduced the severe toxicity of metals and increased the growth parameters. This high efficacy of protection shown in the ecotoxicological models may result from the numerous possible interaction pockets of the β-lactoglobulin-amended materials depicted in simulations. The ecotoxicological models support the agreement with computations. This study serves as a proof of concept on how computations in tandem with experiments can be used in the design of multicomponent clay- and carbon-based sorbent amended systems with augmented functionalities for particular toxins.

## 1 Introduction

The pronounced growth of industrialization and urbanization has significantly contributed to the presence of heavy metal pollution in environmental bodies such as water and soil.^1,2^ Heavy metals can have a substantial environmental impact due to their persistence in the environment and adverse health effects on individuals and biotas.^1,3,4^ Cadmium (Cd) is found in its divalent form in nearly all stable cadmium compounds.^5^ Cd is commonly encountered in a variety of geological materials, including fertilizers, coal, soils, and rocks.^1^ Simultaneously, it plays a significant role in the manufacturing of batteries, pigments, textiles, and metal coatings.^1^ Exposure to Cd primarily arises through dermal absorption, inhalation of contaminated dust, and dietary intake.^6−8^ This exposure is a prominent health concern, associated with breast, kidney, and pancreatic cancer,^9^ hepatic injury, lung damage, and hypertension.^10^ Like Cd, lead (Pb) is usually found in its divalent form, and Pb contamination can stem from industrial activities, such as smelting and mining, as well as the production of consumer products containing lead, such as cosmetics and toys.^11^ Ingestion can result in harmful effects on human and animal health, as Pb can interfere with a variety of bodily systems and functions, such as the nervous and hematopoietic systems, as well as cause adverse renal and cardiovascular effects.^12,13^ Moreover, Pb is considered to be carcinogenic (Group 2B) to humans.^11^

The removal of heavy metals from wastewater remains a challenge. Various techniques have been used to remediate heavy metals including chemical precipitation,^14,15^ coagulation and flocculation,^16,17^ ion exchange,^18^ and ion flotation.^19,20^ However, these techniques are subject to limitations such as high energy requirements, high cost, and limited to processing small volumes of wastewater.^21,22^ Adsorption is an attractive approach for water treatment, particularly because adsorbents are generally cheap, do not require pretreatment before application, and are easy to regenerate.^22,23^

Notably, heavy metal exposure can occur simultaneously with exposure to other toxic substances. For example, the combination of heavy metals, including Cd and Pb, with pesticides has been shown to adversely influence plant and soil health.^24^ Additionally, the cocontamination of soil with Cd, Pb, and polycyclic aromatic hydrocarbons (PAHs) has been reported in industrial settings in multiple environmental media.^25−28^ Thus, it is imperative to consider broad-acting sorbent strategies, involving novel materials, that could accommodate the removal of a wide range of toxins, along with heavy metal ions, that can be present in contaminated water, soil, and food.

Therefore, this study aims to design multicomponent sorbent materials that are inexpensive and environmentally friendly for divalent Cd and Pb, focusing on clay and activated carbon due to their broad-acting sorbent capacities. Clays, such as montmorillonite, have been suggested as promising candidates for edible sorbents due to their ability to bind toxic compounds and heavy metal ions.^29−33^ Montmorillonite clays are promising potential sorbents for multiple toxic compounds including glyphosate, paraquat,^34^ per- and polyfluoroalkyl substances,^29^ aflatoxin,^35,36^ and dieldrin^30^ while an acid-processed montmorillonite clay showed significant reduction (75%) of Pb toxicity.^31^ Additionally, activated carbons are sorbents for a broad range of toxin mixtures, including paraquat, diquat, difenzoquat,^37^ Polychlorinated biphenyls,^38^ and bisphenol A,^39^ while a medical-grade activated carbon showed protection for Cd toxicity.^31^ Nevertheless, there is still a need to develop amended clay- and carbon-based material systems for improved binding and protection against Cd and Pb mixtures.

β-lactoglobulin is the major bovine whey protein, accounting for approximately 10% of the total protein in bovine milk.^40^ The protein has 11 genetic variants, with genetic variants A and B being the most common in bovine milk and differ only in residue positions 64 and 118.^41^ β-lactoglobulin, along with other whey proteins, exhibit a tendency to interact with metal ions.^40^ Previous studies reported β-lactoglobulin’s capacity to interact with different heavy metal ions, including Pd, Co, Ni, and Mn within the context of amyloid–carbon hybrid membranes.^42^ One factor affecting metal–protein interactions is pH, which can change the protonation state of amino acids in the proteins, including Asp, Glu, and His.^40^ Another factor affecting such interactions is temperature, where β-lactoglobulin forms amyloids at higher temperatures (80 °C).^43^ The amyloids formed by β-lactoglobulin were utilized by several studies, in the context of amyloid–carbon hybrid membranes, to absorb heavy metal pollutants from solutions.^44^

Motivated by the ability of β-lactoglobulin to bind particular heavy metal ions, we aimed to engineer montmorillonite clay and activated carbon amended with β-lactoglobulin (in its nonamyloid form) to augment their functional properties for binding Cd and Pb. We considered β-lactoglobulin as a particularly attractive potential amendment due to the fact that it constitutes a safe milk protein, which was found to bind particular metal ions both in its amyloid form^45^ as well as in its nonamyloid form.^40^ Montmorillonite clays and activated carbons are promising broad-acting sorbents for a variety of toxic compounds, and amendments can further expand their functionality, binding, and protection properties, for Cd and Pb. This study was built on our previous studies in which particular amendments were used in conjunction with montmorillonite clays, showing improved sorption properties for toxins,^29,46−49^ as well as on the capacity of MD simulations to study the structure, dynamics, and protein adsorption phenomena to clays^50^ or different surfaces.^51^ The objective of this work was to design multicomponent clay-based and carbon-based amended sorbent systems incorporating amendments to enhance the binding of Cd and Pb in comparison to parent (unamended) material systems, which can potentially be applied in the framework of broad-acting sorbents for multiple toxins.

## 2 Methods

### 2.1 Initial Structure Modeling and Parametrization of a β-Lactoglobulin Dimer and the Materials

The structure of a β-lactoglobulin dimer, variant B, was considered as a means to computationally represent a potential arrangement of two monomers within multimers by the protein.^52,53^ Structures of a β-lactoglobulin dimer, corresponding to pH 3.8 and neutral conditions, were extracted from the PDB, entries 6NKQ chains A and B,^52^5K06 chain A,^53^ respectively. The biological assembly was used to create the protein dimer of 5K06. Unresolved N-terminal residues of the structural conformation selected to initially represent acidic conditions, corresponding to PDB: 6NKQ, were modeled using Swiss PDB Viewer.^54^ The unresolved 109–114 loop in the structural conformation selected to initially representneutral conditions, corresponding to PDB: 5K06, was modeled using SuperLooper2.^55^ PropKa version 2.0^56^ was used to investigate the protonation state of Glu, Asp, and His, and assign corresponding states of these residues at the acidic and neutral conditions in calculations and simulations. Disulfide patches were used to account for disulfide bonds (two for each β-lactoglobulin monomer) within the simulations.^57^ The CHARMM36m force field^58^ was used for protein parametrization in all computational studies.

One layer of calcium montmorillonite clay (CM) was modeled with a composition of (Si_4_)^IV^(Al_1.67_Mg_0.33_)^VI^O_10_(OH)_2_, as in our previous studies.^29,34,47−49,59,60^ CHARMM-GUI^60−64^ was used to initially model the clay with dimensions 50 × 50 Å^2^, Miller indices 001, and a ratio of defect of 0.33333. The modeled clay was considered representative of acidic conditions; INTERFACE FF^62^ provides this clay as a standard for pH 3 (CM-pH3). To model the system at neutral pH 7 (CM-pH7), particular hydrogens and hydroxyl groups from the clay edges were manually removed from the original setup provided by CHARMM-GUI,^60−64^ in line with the clay structures in neutral conditions provided by INTERFACE FF.^62^ Initially, CHARMM-GUI^60−64^ provided two clay layers, and we manually removed one of the layers to model a single layer. Additionally, the initially placed sodium ions provided by CHARMM-GUI^60−64^ were manually deleted as our studies investigated calcium montmorillonite clays. Calcium ions were included in the modeled system in the explicit solvent simulations, in which 20 Ca(II) were placed randomly in the system during the simulation setup, corresponding to the dimensions of the clay layer used, as in our previous studies.^29,34,47−49,59^ In line with CHARMM-GUI,^60−64^ the INTERFACE FF^62^ force field was used for CM parametrization in all computational studies.

One layer of carbon graphene sheet consisting of 350 carbon rings was modeled using CHARMM-GUI,^60,61,63,64^ and two variations with a different amount of defects were considered: 1% (referred to as AC1) and 20% (referred to as AC20), were considered in our effort to account for a small and relatively large number of defects, respectively, which can be present in activated carbon. Both structures comprised both hexagonal and nonhexagonal carbon rings, the latter being more populated in AC20. While the structure of AC1 was relatively flat with a little curvature and contained minimal defects, the structure of AC20 comprised a significant curvature, reminiscent to the curved arrangement of activated carbons depicted in imaging of commercial activated carbon.^65^ According to the literature, activation of carbon yields a fullerene carbon structure, with fragments containing nonhexagonal rings;^66^ in this study, in line with our previous study, we consider that our AC1 and AC20 models could represent these carbon structures.^67^ The same structural models were considered for acidic, pH 3 and neutral pH 7 conditions and are respectively referred to as AC1-pH3, AC20-pH3, and AC1-pH7, AC20-pH7. Structure and parametrization files for carbon materials, AC1 and AC20, were produced through CHARMM-GUI.^60,61,63,64,68^

### 2.2 Computational Docking Studies of a β-Lactoglobulin Dimer onto the Material Layers

We introduced six independent docking rounds using in-house CHARMM programs of the protein dimer in complex with (a) CM-pH3, (b) CM-pH7, (c) AC1-pH3, (d) AC1-pH7, (e) AC20-pH3, and (f) AC20-pH7. Our procedure for docking allowed a nearly exhaustive search of the protein dimer orientation to the materials under investigation. To achieve this, our procedure consisted of 1,000 rounds of docking runs per case,^69^ aiming at efficient exploration of protein dimer binding poses. For each round, the following steps were performed: The protein dimer and the material were centered, followed by a translation of the protein dimer along the *z*-axis away from the material, given that the material was on the *x*–*y* plane. The protein dimer was then rotated 90° around a randomly generated axis, which ensured a random orientation of the protein dimer. Subsequently, an energy minimization was performed subject to constraints, allowing the protein dimer to approach the material. During the minimization, the material was fixed, and bestfit constraints were applied to the protein dimer to preserve the integrity of the protein dimer. In each case, the resulting binding pose of the protein dimer to the material was recorded for further analysis in what follows.

The produced docking configurations for the material systems (materials amended with a β-lactoglobulin dimer) were scored using interaction energy calculations in CHARMM^63^ considering the sum of van der Waals (vdw) and electrostatic contributions in all systems.^69^ We additionally used DLIGAND2^70^ in systems involving carbon materials (c–f); in this way, each carbon layer was considered as a “ligand” binding to a protein, as an additional metric to assess the docking modes. In systems (a) and (b), the docked complex structures with the lowest interaction energy were extracted as initial conformations for further investigation within the MD simulations. In systems (c–f), a consensus ranking was employed to select the docked structures. This was done by ranking the five structures with the lowest interaction energy and the five structures with the lowest DLIGAND2^70^ energies, creating an overall rank. In the end, we selected and derived the structure with the lowest overall summed rank for further investigation using MD simulations (see below). It is worth mentioning that the protein dimer was in the same orientation for the top three ranked structures for all material systems.

### 2.3 Initial Introduction of the Heavy Metal Ions in the Modeled Proteins

Cd and Pb ions were initially introduced individually to the modeled protein dimers using MIB2, a metal ion-binding prediction server.^71^ The server results were refined so that any ions within 6.5 Å of another predicted ion were excluded, taking the higher-scoring ion and removing the lower-scored ion. Additionally, to ensure consistency in the ion placement in subsequent simulations, we compared the placements of Pb and Cd. All Pb binding pockets were represented in the Cd binding pockets, but not all of the Cd binding pockets were represented by Pb binding pockets. Therefore, additional Pb ions were introduced, where the server only predicted Cd binding. We considered that any unfavorable binding of ions would be indicated within the molecular dynamics (MD) simulations that were subsequently performed. This resulted in 21 placed Cd and Pb ions, independently, in complex with the modeled protein dimers at both acidic and neutral conditions and was replicated accordingly to the docked structures. It is important to note that, to our understanding, the server could not differentiate between different pH conditions; thus, we used the same initial placement for the two cases and considered that the favorability of binding of the ions at different pH conditions could be examined more thoroughly using simulations. Indeed, as observed below, the MD simulations were able to capture the loss of binding to pockets consisting of protonated carboxyl groups of aspartic and glutamic acids, which corresponded to acidic conditions.

Additionally, we aimed to investigate systems in which ions were not placed initially by the server but instead investigate ions that were randomly initially distributed (e.g., in the simulation box; see below). Thus, 10 Cd or Pb ions were randomly placed in all of the corresponding systems (without any overlap with the protein dimer proteins in complex with the materials). This aimed at exploring and investigating other potential binding pockets of the two heavy metal ions, presumably formed between the proteins and the materials, and which were not necessarily predicted by the MIB2^71^ server.

### 2.4 Simulation Setup

The selected docked (protein dimer-material complex) structures for all systems^69^ were used as initial configurations within the simulation setup to study each corresponding system. The procedure was primarily based upon CHARMM-GUI^60−64^ with particular modifications, as presented in what follows. Each modeled system was initially centered in a cubic 100 Å periodic boundary conditions box, solvated by explicit TIP3P water molecules in CHARMM,^63^ and neutralized using chloride ions. Parameters for Cd and Pb^72^ were given by CHARMM-GUI.^60−64^ Prior to the MD simulations, the modeled systems were minimized (50 steps of SD/50 steps of ABNR) and equilibrated at constant volume for 2 ns in CHARMM,^63^ and the production MD simulations were run using OPENMM.^73^ A constraint of 0.956 kcal mol^–1^ Å^–2^ (400 kJ mol^–1^ nm^–2^) was applied to the Mg and Al atoms of the clay during equilibration and production, for the CM systems, as in.^47,67,74^ For each system, either with server-placed ions or randomly placed ions, triplicate runs starting from the corresponding initial configurations were performed at a constant temperature of 300 K and constant pressure (1 atm), and each ran for 100 ns. A uniform isotropic barostat was used in all of the simulations. For each of the following systems, (a) CM-pH3, (b) CM-pH7, (c) AC1-pH3, (d) AC1-pH7, (e) AC20-pH3, and (f) AC20-pH7, we investigated the binding of both Cd and Pb, independently, starting from ions initially placed on the basis of server predictions or ions initially placed randomly in the box. Upon completion of the triplicate runs for the 24 different initial setups, snapshots were extracted every 1 ns for all systems and used for analysis.

### 2.5 Computational Analysis of Simulation Trajectories

After completion of the MD simulations, a combination of in-house FORTRAN and Python programs were developed to investigate the binding capacity of the protein dimer to the material, as well as the binding propensity of both heavy metal ions to the β-lactoglobulin amended material systems. After visual inspection of the material systems, we considered the first 30 ns of the simulation as an additional equilibration stage to account for the stabilization of binding pockets within the system and thus were excluded from the analysis, leaving the last 70 ns of the simulations of each triplicate run to be examined by the analysis programs, and analysis was performed every 1 ns. In the analysis, we used a rather relaxed distance criterion of 5.0 Å cutoff to characterize interactions between heavy atoms of different entities and focused on protein-ion, protein-material, and ion-material interactions. The criterion facilitated the characterization of interactions between heavy metal ions and other entities and considered effectively the fluctuations between different protein amino acids with the heavy metal ions. According to our programs, if any pair of heavy atoms between two interacting entities (e.g., an amino acid of a protein and the heavy metal ion, an amino acid of a protein and any atom of the material, and the heavy metal ion with any atom of the material, respectively) was within 5.0 Å, then, according to our definitions, the two entities interact. VMD was used to visualize simulation snapshots.^75^

First, we investigated the capacity of proteins to remain in contact with the materials leading to the formation of the material systems (i.e., protein-amended CM and protein-amended AC) by studying the percent probability of the protein dimer to interact with the material throughout the simulations. The percent probability was normalized by the total number of residues in the protein dimer and the number of snapshots. We additionally traced the average number of bound residues from both proteins independently to each of the materials. Additionally, we performed various root-mean-square deviation (RMSD) calculations of the protein backbone (N, Cα, and C) for each simulation trajectory. We initially calculated the average RMSD values for each of the simulation trajectories and reported the statistical average and standard deviation across all simulations of the same material systems. The first RMSD calculation, termed “alignment with respect to (wrt) initial structure”, was performed by aligning the simulation conformations of the protein dimer with respect to the initial structure of the protein dimer of each trajectory; this reflects a measure of the conformational change of the protein dimer within the simulation with respect to their initial structures. The second RMSD calculation, termed “no alignment”, was performed without any alignment of the simulation conformations for the protein dimer; this reflects a measure of the change in protein dimer position (translation and rotation) and conformation within the simulation with respect to the initial structure in each simulation. For the CM simulation trajectories, due to the constraints imposed on the Mg and Al atoms during the simulation, no additional superposition of the clay was necessary. For AC systems, a superposition of carbon atoms was performed, as the carbon was allowed to freely move during the simulation. The third RMSD calculation, referred to as “no alignment with respect to average structure”, was performed as the second RMSD calculation, with the difference that the reference structure used for each calculation corresponded to the average structure per trajectory; this reflects a measure of the change in protein dimer position (translation and rotation) and conformation within the simulation with respect to the average structure in each simulation. A consensus of (i) the lowest RMSD value, in conjunction with (ii) the highest percent probability of the protein dimer interacting with the material, was used to select the trajectory with the most “successfully formed” material system for each case (i.e., the trajectory per system with the lowest RMSD value and the highest average). For each of the selected trajectories per case, we reported the type of amino acids bound to the materials, as well as reported analytically bound residues from the two proteins, within the last simulation snapshot; in this analysis, Cd and Pb, despite being two independent cases, were combined together, as at this stage, emphasis was given on elucidating the properties of the material systems rather than the heavy metal ion binding properties.

Second, we investigated the propensities of the two heavy metal ions to be bound to the material systems with either server-placed ions or randomly placed ions; importantly, here, Cd and Pb were considered independently. We determined the overall binding propensity of the heavy metal ions to interact with each of the material systems. We categorized binding events of the heavy metal ions to the material systems into three modes: (i) the heavy metal ion to be bound to both the material and one or both proteins simultaneously, (ii) the heavy metal ion to be bound to one or both proteins (and not the material),^63^ the heavy metal ion to be bound to the material only. For each mode in categories (i) and (ii), we aimed to elucidate the key constituent protein residues composing the ion pockets, in our effort to uncover the predominant binding pockets for each case and ultimately compare against (a) different material systems, (b) different pH conditions, and (c) and different ions (Cd or Pb). Due to the fluctuations of the residues involved in each ion’s binding within the simulations, we aimed to identify a consensus binding pocket that is sufficiently well preserved throughout the simulation by applying the following: A binding pocket for a particular ion should have been present in at least 60% of the analyzed simulation snapshots per trajectory in at least one trajectory per case and should have been maintained in the corresponding trajectory last simulation snapshot; additionally, the corresponding residues in the pocket reported are the ones which interacted with the heavy metal ion in at least 50% of the instances that the pocket was formed.

### 2.6 Experimental Methods on Materials, Sorbent Synthesis and Characterization

Metal chlorides of Cd and Pb for use in toxicity studies were obtained from Sigma Chemical Company. Calcium montmorillonite (CM) clay was obtained from BASF (Ludwigshafen, Germany), and the medical grade powdered active carbon (AC), purity >99%, was obtained from General Carbon Corporation (Paterson, NJ). Clays and carbons were sieved at 100 mesh to achieve a uniform particle size (≤149 μm). The physicochemical properties of these base materials have been characterized.^76,77^ β-lactoglobulin was purchased from Sigma-Aldrich (St. Louis, MO) and stored at 4 °C. A previous method^44^ was followed to yield protein hybrid membranes, where 20 mL of a 10% dispersion of AC and CM was separately mixed with 2 mL of a 2% β-lactoglobulin protein solution at pH2 for 1 h at ambient temperature. The solution was filtered through 0.22 μm cellulose filters (diameter of 25 mm) and dried in a desiccator. An aggregation assay^78^ was conducted using Amicon filters (50 kDa cutoff) and identified that 62% of the amended β-lactoglobulin were soluble (dimers and trimers) and the rest were aggregates (tetramers and bigger).

The zeta potential and particle (hydrodynamic) size of 1 mg/mL CM-lactoglobulin and AC-lactoglobulin suspensions were measured three times with 13 runs each time by a Zetasizer Nano ZC (Malvern, UK) at 25 °C. The synthesized sorbents were also characterized by a field emission scanning electron microscope (SEM, JSM-7500F, JEOL, Peabody, MA) with 5 nm of Pt–Pb coating and Fourier-transform infrared spectroscopy (FTIR, IRPrestige-21, Shimadzu, Japan). The external surface area was calculated by the Brunauer–Emmett–Teller (BET) method with nitrogen absorption using a Micromeritics 3Flex Adsorption Analyzer (Norcross, GA). All samples were activated at 200 °C for 4 h before assessing the absorption of nitrogen at 77 K.

### 2.7 Ecotoxicological Models

*Lemna minor* (duckweed) was purchased from AquaHabit (Chatham, England). The plant was cultured in growth media with cool white fluorescent lights (400 ft-c intensity) at a light-to-dark cycle of 16 h/8 h and an ambient temperature of 25 °C. Three colonies of 3-frond lemna plants were randomly selected and incubated in Pyrex dishes closed with loose-fitting lids for 7 days. Lemna was exposed to varying doses of Cd and Pb chloride and a mixture of Cd and Pb chloride from 0.1, 0.2, 0.4, 0.8, and 1.6 ppm (μg/mL) to determine the dose that resulted in more than 50% of reduction in all growth parameters. For the detoxification study, metals were treated with 0.05 and 0.1% CM-lactoglobulin and AC-lactoglobulin for 7 days, with comparison to the base CM and AC. Lemna was inspected daily for the frond number and the surface area of surviving plants, which was analyzed by ImageJ (NIH, Bethesda, MD). On day 7, the plants were removed from individual dishes and homogenized in 1.5 mL of 80% acetone. The chlorophyll content was extracted after 48 h (4 °C, dark) and measured by UV–vis scanning spectrophotometry (Shimadzu UV-1800, Kyoto, Japan) at 663 nm.

*Hydra vulgaris* were obtained from Environment Canada (Montreal, QC) and maintained at 18 °C and neutral pH. Using a hydra classification method,^79^ the morphology of the hydra was rated on a 10–0 point scale as an indicator of toxicity, where a score of 10 represented normal, healthy hydra and a score of 0 represented disintegrated hydra. Three hydra colonies were included in each group and exposed to 4 mL of test media in Pyrex dishes. The average score for each group was used to determine the toxicity rating at each time point (0, 4, 20, 28, 44, 68, and 92 h). Doses of metals that resulted in more than 50% morphological degradation (score <5) in 92 h were identified as 5 ppm (μg/mL) Cd, 15 ppm Pb, and 3 ppm of a mixture of Cd and Pb. These concentrations were included in the detoxification study and treated with sorbents including CM-lactoglobulin and AC-lactoglobulin at 0.05, 0.1, and 0.2% inclusion rates in the hydra media, with comparison to the base CM and AC.

Each group consisted of 3 colonies and was conducted in triplicate for various end points. Data significance was verified by the analysis of variance following Dunnett’s *t* test. Significance was set at *p* ≤ 0.05.

## 3 Results and Discussion

### 3.1 Binding Capacity between the β-Lactoglobulin Protein Dimers and the Materials

**Figure 1:**
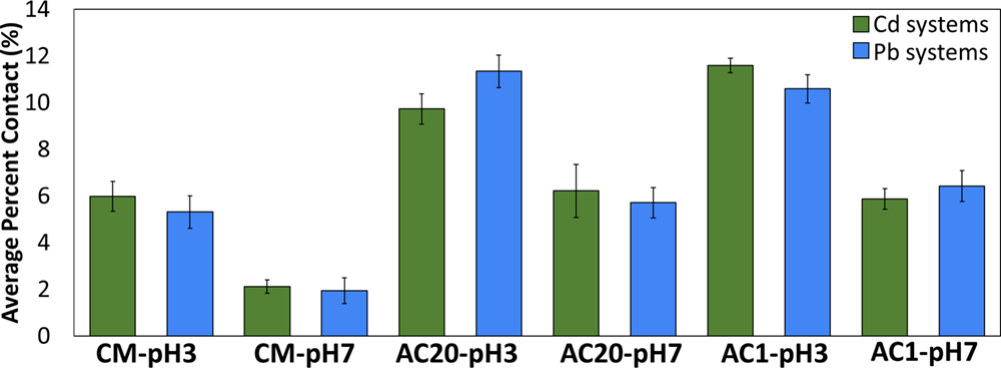
Average percentage probability of contact between a protein dimer and each of the corresponding materials. Statistics for average and standard deviation values were calculated over all trajectories. Green bars represent Cd systems, and blue bars represent Pb systems.

Within all of the simulations, the β-lactoglobulin protein dimer remained in contact with the materials (CM, AC1, and AC20) for both pH conditions, and the percent contact probability was plotted for all material systems. Figure 1 shows the average percent contact of the protein to the material for each system throughout the simulation. The large surface area of the modeled activated carbon, especially AC20, provided grooves that can accommodate multiple binding sites for the protein. In addition, AC1 and AC20, provide an aromatic-/hydrophobic-rich surface which can enable different types of interactions with protein residues, including hydrophobic, aromatic, and positively charged residues; the latter can form cation-π interactions (see below). As a result, AC1 and AC20 acquired an overall higher percent contact probability compared to CM-pH3 and CM-pH7, with no significant differences between AC1 and AC20 (Figure 1).

**Figure 2:**
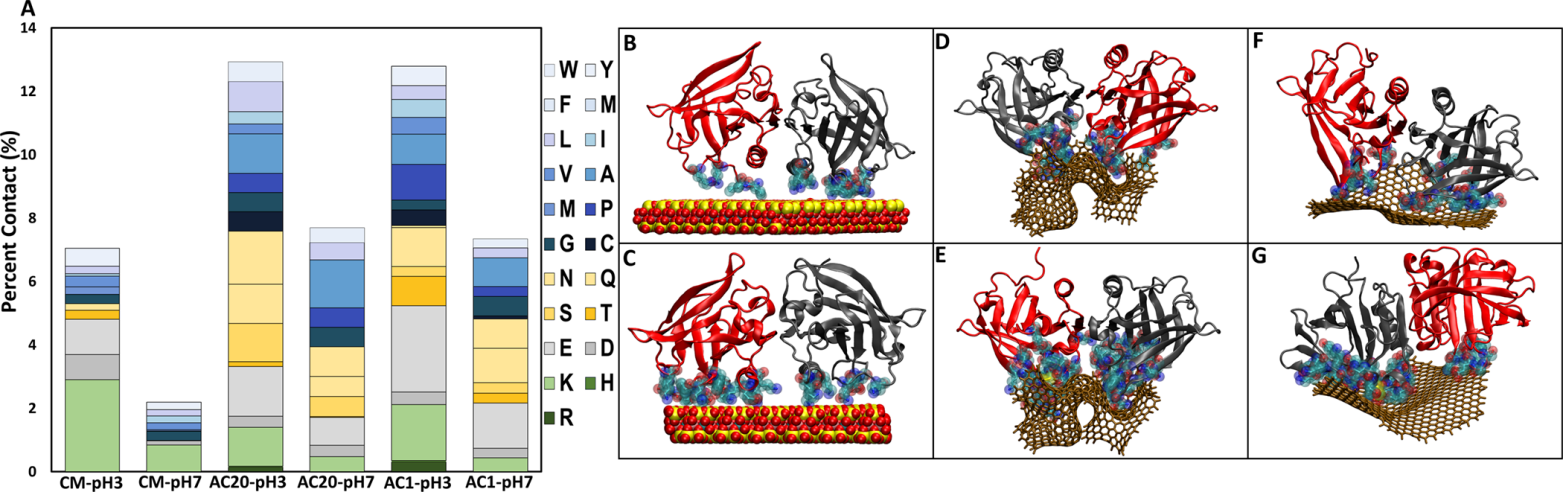
(A) Percent contact of the protein dimer with the materials. When the protein is in contact, the colors within the bars represent which type of amino acid is in the interface (adding 100% to the contacts). R, H and K are colored green-like, D and E are colored gray-like, T, S, Q, N are colored yellow-like, C, G, P, M, A, V, I, L, M, F, Y, W are colored blue-like. (B–G) Visual presentation of the corresponding final simulation snapshot of selected trajectories, per system (B) CM-pH7, (C) CM-pH3, (D) AC20-pH7, (E) AC20-pH3, (F) AC1-pH7, and (G) AC1-pH3. The β-lactoglobulin dimer is depicted as a new cartoon diagram, with one monomer in red and the other in place. Side chains of contacting amino acids are shown in vdW.

Additionally, the percent contact probability was higher under acidic conditions than under neutral conditions across all different systems (Figure 1). This can be partly attributed to the presence of fewer negatively charged residues at lower pH conditions at the binding interface, by protonated Asp and Glu residues. At neutral conditions, Asp and Glu contributed to salt bridge formation with oppositely charged amino acids, and the latter contributed less to the binding, for CM (comparing CM-pH3 vs CM-pH7), AC1 (comparing AC1-pH3 vs AC1-pH7), and AC20 (comparing AC20-pH3 vs AC20-pH7), as seen in both Figures 1 and 2. Figure 2A illustrates the percent contribution per residue type in the formation of contacts between the protein dimers and the materials for the last simulation snapshot in selected trajectories (see Methods). Figures 2B–G present visual representations of the protein dimer in complex with the materials, with the involved amino acids highlighted, for last simulation snapshot in the selected trajectories (see Methods). These figures provide an illustration of how aromatic-/hydrophobic-rich surfaces in AC20 and AC1 facilitate interactions with a diversity of protein residues, in contrast to CM for which most contacts were formed by positively charged residues, predominantly Lys.

**Figure 3:**
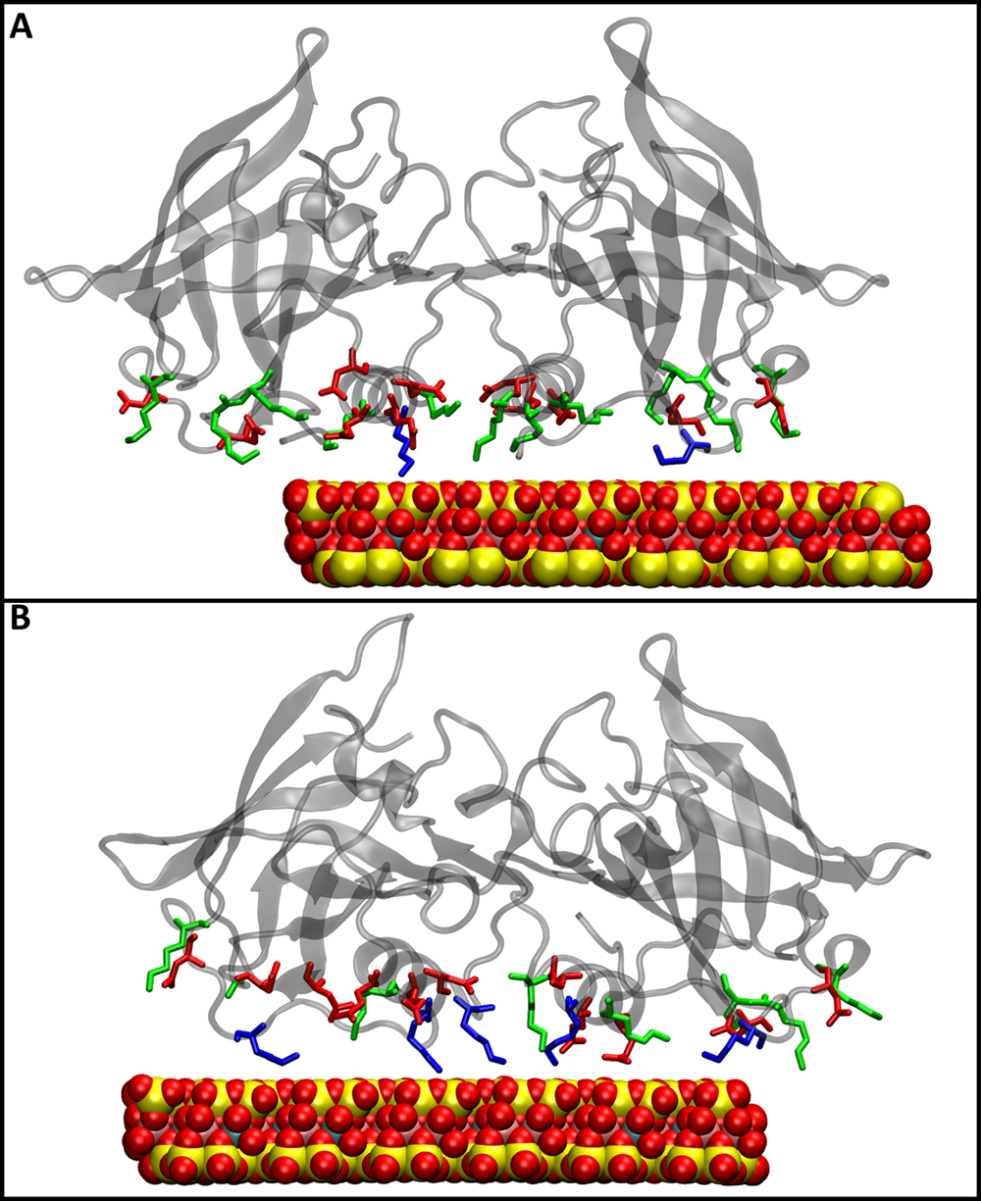
Simulation snapshot of selected trajectories showing salt bridges formed in (A) CM-pH3 and (B) CM-pH7. Bound Lys residues are represented in blue licorice representation, while unbound Lys residues that are interacting with Asp or Glu residues are shown in green licorice representation. Asp and Glu residues are shown in red licorice representation. CM is shown in vdw representation. The protein dimer is shown in transparent new cartoon representation.

Additionally, for CM systems, at acidic conditions (CM-pH3), the absence of charge for Asp and Glu residues, which are in proximity to Lys residues, allowed the latter to be solvent exposed, facilitating contacts and interactions with the clay (Figure 3A). For neutral conditions (pH7), the corresponding Asp and Glu residues were charged; their capacity to form salt bridges with Lys residues weakens the capacity of Lys residues to interact with the clay (Figure 3B). Lys residues were the main contributors of protein binding to the material, indicated by the percent contact of the protein dimers with the materials decomposed by the amino acid type and visualization of the simulation snapshots (Figure 2A, B). However, the number of Lys residues not bound to the material was higher in CM-pH7 compared to CM-pH3, which can be attributed to their interaction with Asp and Glu residues and the formation of intramolecular salt bridges. Nevertheless, we observed that interactions were sufficient for material systems to be formed, and consequently, β-lactoglobulin protein dimers to be amended to clays. It is also important to note the difference in protein dimer orientation within the various material systems. In CM systems, the protein dimer was oriented with its helixes down toward the material (Figure 2B, C). This differs from AC20 systems (Figure 2D, E), where the protein dimer is flipped with respect to its orientation in CM systems, and the helices are up, oriented away from the material. In AC1 systems, the helixes were neither oriented up nor down, but rather more on the side in reference to the material (Figure 2F,G). The protein dimer orientation did not appear to play a major role in binding pockets formed to bind to the two heavy metal ions (see below).

RMSD calculations were performed to assess the change in protein dimer conformation and position within the simulations with respect to the initial or average structure used in each case. Table 1 lists both average and standard deviation RMSD values across all simulation trajectories of a material system as well as the corresponding values for the aforementioned selected systems. The overall conformation of the protein dimers was fairly maintained with respect to the initial conformations, as depicted by the relatively small RMSD calculations presented in the second column of Table 1. RMSD values showing the combined changes in protein dimer conformation and position (translation and rotation) within the simulation with respect to the initial structure are presented in the third column of Table 1. They overall reflect that, even after the first 30 ns, which was considered as additional equilibration time, the proteins tended to continue adapting their position on the material, which seemed to reduce over time. The latter is depicted by the smaller RMSD values in the fourth column of Table 1, showing that systems overall, and especially selected trajectories in each of the systems, have smaller deviations from their positions and orientations with respect to their average structure. Smaller values were calculated for AC vs CM, and for CM-pH7 vs CM-pH2, which correlate with our aforementioned observations on the contacts formed between the protein and the corresponding material systems. Particularly, there was an overall correlation between a decreased number of contacts and a larger RMSD of the protein dimer. The largest value across the selected systems (4.78 Å), corresponding to CM-pH7, was primarily indicative of higher variability of the protein dimer position owing to the smaller number of contacts to CM compared to the other systems. Yet, it is important to highlight that since no alignment on the protein backbone was performed and given that this value also encompasses changes in the intrinsic conformational variability of the dimer, the material appears to provide at least some stability for the protein dimer in the system with the fewer number of contacts between the protein dimer and the material. Importantly, in all other cases, the stability of the material-protein systems is higher.

**Table 1:** Column 1: Material Systems, Columns 2, 3, and 4 Correspond to “Alignment wrt Initial Structure”, “No Alignment”, and “No Alignment wrt Average Structures”, Shown in Methods^a^

systems	RMSD values		
	alignment wrt initial structure	no alignment	no alignment wtr average structure
CM-pH2	1.71 ± 0.36 (1.69)	6.23 ± 2.25 (2.79)	3.54 ± 1.51 (1.70)
CM-pH7	2.21 ± 0.43 (1.52)	9.65 ± 4.65 (5.50)	5.76 ± 1.67 (4.78)
AC1-pH2	1.90 ± 0.35 (1.79)	4.07 ± 2.17 (2.09)	2.87 ± 1.74 (1.79)
AC1-pH7	2.84 ± 1.36 (1.38)	6.65 ± 2.96 (3.61)	4.04 ± 1.80 (2.57)
AC20-pH2	1.91 ± 0.72 (2.05)	3.16 ± 1.83 (1.77)	2.16 ± 1.39 (1.27)
AC20-pH7	1.97 ± 0.36 (1.62)	5.38 ± 1.73 (2.85)	3.30 ± 1.42 (1.77)

a Values outside parentheses correspond to the average RMSD across six runs per system, and the standard deviation is calculated for the six corresponding averages. Values in parentheses denote the corresponding RMSD for the particular selected system per case. All values reported are in Å.

### 3.2 Binding Propensities of Cd and Pb to the Material Systems

**Figure 4:**
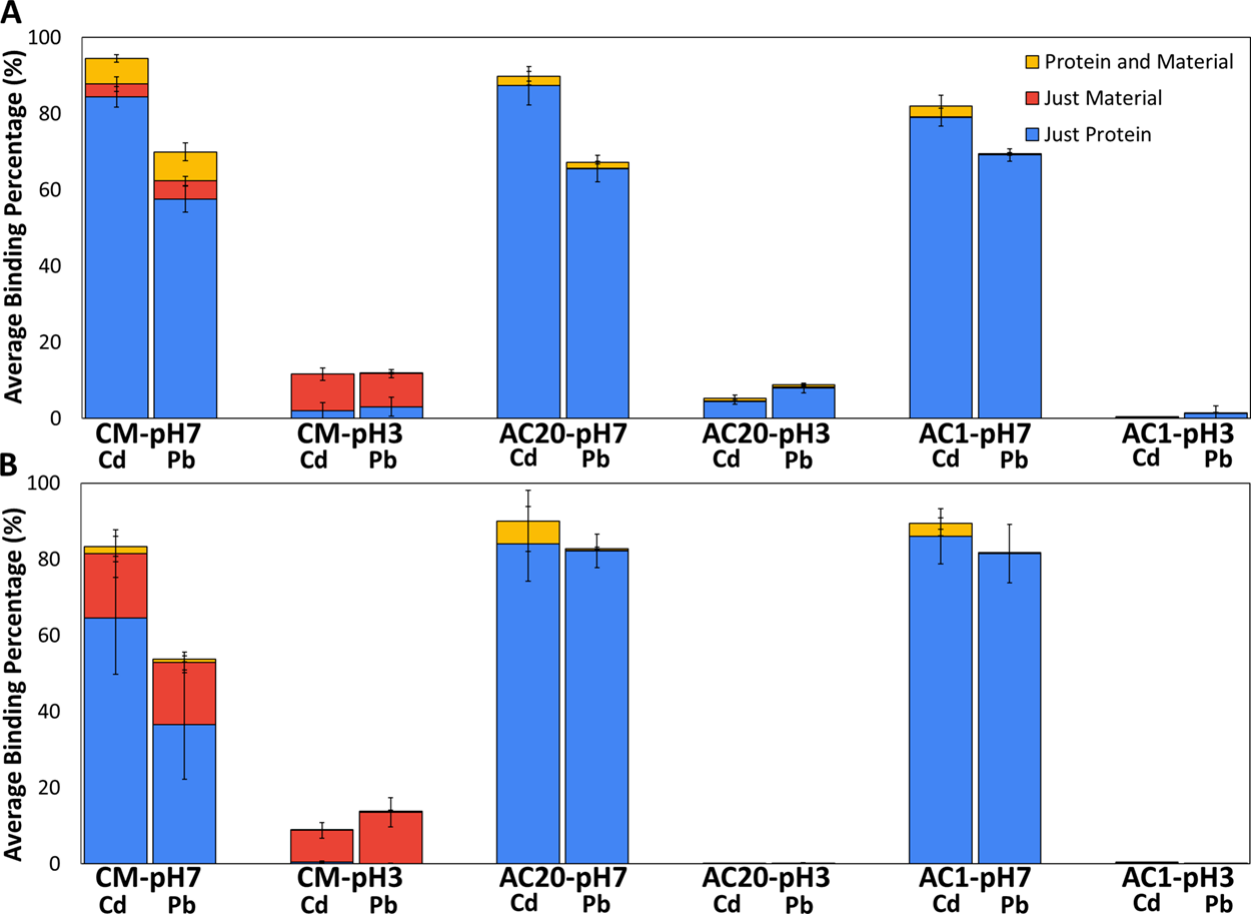
Overall binding of heavy metal ions, Cd and Pb, for the various material systems in (A) systems of server-placed ions and (B) systems of randomly placed ions. Blue columns represent ion-protein binding, red columns represent ion-material binding, and yellow columns represent ion-material-protein binding.

We calculated the percentage probability of the two heavy metal ions binding to the amended materials within the simulations, distinguishing between binding to the protein dimer only, binding to the material only, and concurrent binding to the protein dimer and the material. We observed that both Cd and Pb ions had a high binding propensity to the material systems at pH 7, irrespective of whether the heavy metal ions were initially placed on the basis of server predictions (Figure 4A) or if ions were initially placed randomly in the box (Figure 4B). This interestingly shows that simulations effectively provided the capacity for ions to freely move during the simulations and bind to the material systems in the latter. Overall, the material systems at pH 7 had a relatively high binding propensity to the two heavy metal ions, regardless of the initial placement of the ions.

In all material systems, the binding propensity of Cd was slightly higher than that of Pb, and overall the binding of each heavy metal ion independently across different material systems was comparable. In addition, in CM systems, there was a higher percentage of material-ion binding, than in the AC systems, represented by the red color in Figure 4. In CM systems, both heavy metal ions could bind at both the edges and the surface of the material, according to visual observation. It is also important to note that heavy metal ion binding probability to the protein dimer at neutral conditions was far higher than heavy metal ion binding at acidic conditions, which can be attributed to the negative charge of particular Asp and Glu residues coordinating with the heavy metal ions at neutral conditions (see below).

### 3.3 Binding Pockets of Cd and Pb within the Material Systems at Neutral Conditions

In what follows, our analysis focused on elucidating the binding mechanism(s) of Cd and Pb within CM-pH7, AC20-pH7, and AC1-pH7. The high binding propensity of both Cd and Pb to these material systems could be attributed to several binding pockets that can be formed by the protein dimer, facilitating stable binding of the two ions. Simulations revealed the potential for consistent binding to binding pockets created at the protein dimer interface and the interface between the protein and base materials.

Table 2 presents the various protein dimer binding pockets that were identified and maintained in the simulations, for each material system and ion type, according to the criteria set for the identification of binding pockets described in Methods. In Table 2, the first column names each of the binding pockets for indexing purposes. The second column defines the binding pockets formed by the protein dimer residues that were identified in the simulations, while it also reports additional protein residues in parentheses that were observed in particular systems only, according to the criteria established in Methods. These protein residues are shown in the corresponding systems in which they were observed. It is also important to note that no binding pockets were sustained in acidic conditions, and thus, this section focused only on CM-pH7, AC20-pH7, and AC1-pH7 (i.e., systems in neutral conditions). A zoomed-out figure of the binding pockets for CM-pH7, AC1-pH7, and AC20-pH7 are shown in Figures S1–S3, respectively.

**Table 2:**
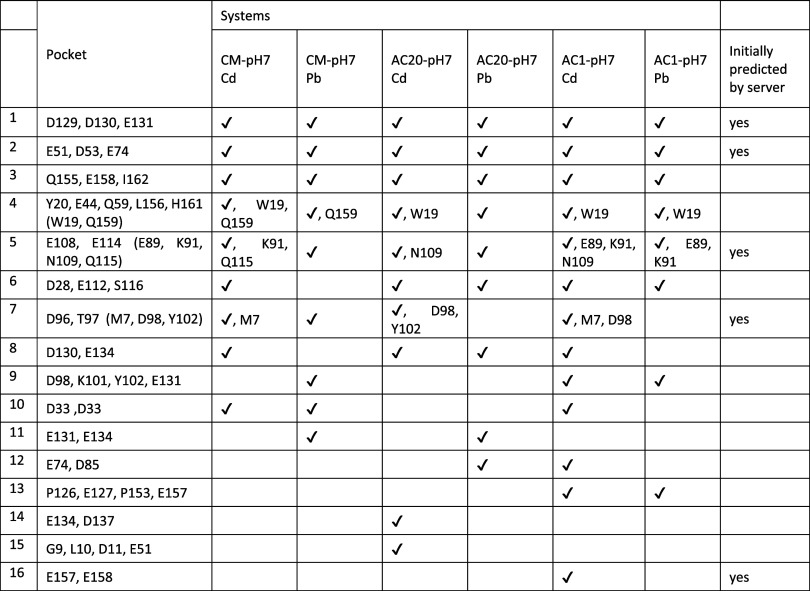
Binding Pockets Were Identified within the Simulation Trajectories^a^

a Column one numbers each of the binding pockets. Column two lists protein residues that were identified to be binding pockets and lists additional protein residues in parentheses that were identified to be specific residues that appeared in only certain systems. The specific residues are reported in the column of the respective system in which they were observed. Columns three to eight refer to the different material systems and the ion types. Column nine identifies binding pockets that were initially predicted by the server. Check marks denote the full binding pocket appearing in the respective systems (residues not in parentheses).

**Figure 5:**
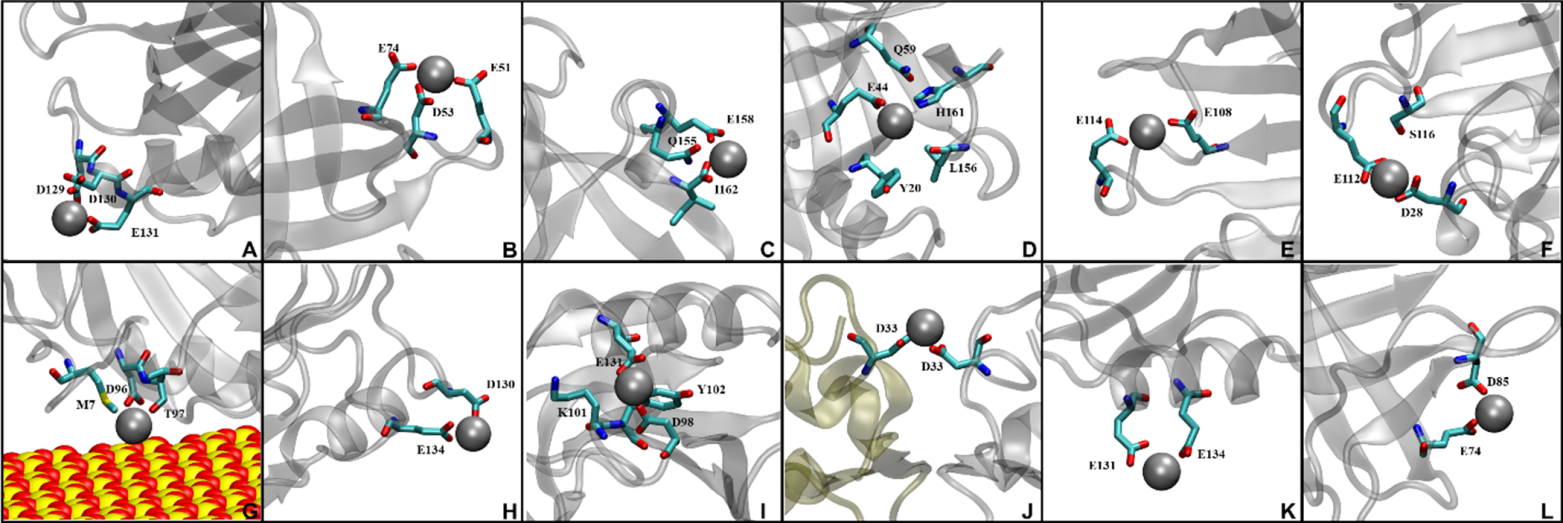
Simulation snapshots of the binding pockets present within the simulation. Active protein residues are shown in licorice, heavy metal ions are shown in vdw, and the protein is shown in new cartoon representation. Monomers of the protein are distinguished by different colors. Corresponding pockets are as follows: (A) D129, D130, E131, (B) E51, D53, E74, (C) Q155, E158, I162, (D) Y20, E44, Q59, L156, H161, (E) E108, E114, (F) D28, E112, S116, (G) M7, D96, T97, CM, (H) D130, E134, (I) D98, K101, Y102, E131 (J) D33, D33, (K) E131, E134, and (L) E74, D85.

The first (1) and second (2) binding pockets comprised residues D129, D130, E131, and E51, D53, E74, respectively, and were identified in all the material systems, as denoted in Table 2. Representative binding pockets 1 and 2 observed within CM-pH7 are shown in Figure 5A and B, respectively. The third (3) binding pocket comprised residues Q155, E158, and I162 and was identified in all material systems. In contrast to the majority of pockets comprising at least two negatively charged residues, in this case, the negatively charged carboxylic C-terminus of I162, instead, participated in this pocket. A representative binding pocket 3, observed within CM-pH7, is shown in Figure 5C. The fourth (4) binding pocket comprised residues Y20, E44, Q59, L156, and H161 and was identified in all material systems, with particular variations. The pocket comprised an additional residue W19 in CM-pH7 for Cd(II), AC20-pH7 for Cd(II), and AC1-pH7 for both ion types, as well as Q159, in CM-pH7 systems for both ion types. Interestingly, the binding pocket included protein residues His-Glu-Gln interacting with the heavy metal ions, which is in line with previous studies for Cd^80,81^ and Pb^82^ showing coordination of these residues with the heavy metal ions. A representative binding pocket 4, observed within CM-pH7, is shown in Figure 5D. The fifth (5) binding pocket comprised residues E108 and E114 and was identified in all material systems, also with particular variations. The pocket comprised additional residues E89 for AC1-pH7 systems, K91 for the CM-pH7 systems for Cd as well as AC1-pH7 systems, N109 for AC20-pH7 systems for Pb and AC1-pH7 systems for Cd, and Q115 for CM-pH7 systems for Cd. A representative binding pocket 5, observed within CM-pH7, is shown in Figure 5E. The sixth (6) binding pocket comprised residues D28, E112, and S116, and was present in all material systems, except it was not observed in CM-pH7 for Pb. A representative binding pocket 5, observed within CM-pH7, is shown in Figure 5F. The seventh^83^ binding pocket comprised residues D96 and T97 and was identified, with variations, in all material systems, with the exception of AC20-pH7 for Pb and AC1-pH7 for Pb. For Cd, the pocket also included M7 for CM-pH7 and AC1-pH7, D98 for AC20-pH7 and AC1-pH7, and Y102 for AC20-pH7. Interestingly, this binding pocket also is reminiscent of the binding pocket that Pt-containing cisplatin forms with β-lactoglobulin.^84^ According to this experimentally resolved pocket, the platinum is in proximity to M7 and D96.^84^ It is important to note that this pocket did appear in both simulation types (server-placed ions vs randomly placed ions) for CM-pH7 for Cd, which highlights the ability of the methods used to not only reproduce but also capture this interaction. Interestingly, a similar binding pocket was identified by Balasco et al.^84^ for Pt, as part of cisplatin, in its interaction with β-lactoglobulin. While it appears that this pocket can be formed and maintained irrespective of the material (CM or AC), it appears that clay could enable this interaction, and a representative binding pocket 7 is shown for CM-pH7 in Figure 5G. The particular pocket does not involve any mediation by carbon materials; as in both AC1 and AC20, this pocket was not proximal to the material. The eighth (8) binding pocket comprised residues D130 and E134, and was identified in all material systems, but was not observed in CM-pH7 for Pb or AC1-pH7 for Pb, and the ninth^83^ binding pocket comprised residues D98, K101, Y102, and E131, and was only identified in CM-pH7 for Pb and for AC1-pH7 for both ion types. Representative binding pockets 8 and 9 observed within AC1-pH7 are shown in Figure 5H, I, respectively. The tenth (10) binding pocket comprised residue D33 from both protein dimers and was identified in CM-pH7 for both ion types and AC1-pH7 for Cd. A representative binding pocket 10 observed within CM-pH7 is shown in Figure 5J. The eleventh (11) binding pocket comprised residues E131 and E134, and was only identified in the CM-pH7 system for Pb as well as AC20-pH7 for Pb. A representative binding pocket 11 observed within AC20-pH7 is shown in Figure 5K. The twelfth (12) binding pocket comprised residues E74 and D85 and was only identified in AC20-pH7 for Pb and AC1-pH7 for Cd. A representative binding pocket 12 observed within AC1-pH7 is shown in Figure 5L.

Lastly, Table 2 also reports less commonly occurring binding pockets, including binding pocket thirteen (13), comprising protein residues P126, E127, P153, E157, binding pocket fourteen (14), composed of protein residues E134, D137, binding pocket fifteen (15), comprising protein residues G9, L10, D11, E51, and binding pocket 16 (16), composed of protein residues E157, E158.

Additionally, it is worth mentioning the variations between simulations of server-placed ions and simulations of randomly placed ions. For each of the binding pockets listed in Table 2, for Cd and Pb independently, we calculated the corresponding occurrence percentage decomposed for each material type Figures S4–S6 decomposed to server-placed ions versus simulations of randomly placed ions. Figures S4–S6 correspond respectively to CM-pH7, AC20-pH7, and AC1-pH7 systems. The diversity of binding pockets observed with the simulations stemming from server predictions was higher than the simulations in which the ions were initially randomly placed and allowed to freely interact with the protein. This is not surprising, as it could be attributed to the fact that in the former case, numerous binding pockets were predetermined by the server, and the simulations were mostly used to examine the capacity of the heavy metal ions to possess or not stable binding at the pockets, under the particular conditions and systems investigated. Nevertheless, it is also important to note that interestingly, in the latter case, simulations provided the opportunity for the formation of particular binding pockets, reminiscent of particular ones also predicted by the server. Importantly, we would like to highlight the advantage of both simulation setups, providing us with an important wealth of knowledge on how heavy metal ions can be recognized by the particular material systems and the conditions investigated, both using the partly biased initial position of ions or not. In other words, both methods were used to amplify the sampling and investigate possible mechanisms of binding of the heavy metal ions to the material systems, rather than to investigate differences and similarities by comparing among the two.

### 3.4 Experimental Sorbent Characterization

To validate metal interactions with β-lactoglobulin predicted in computational simulations above, the β-lactoglobulin protein was amended and characterized on CM and AC structures. As shown by the SEM image, CM-lactoglobulin (β-lactoglobulin amended CM) maintained the typical layer-lattice structure of montmorillonite clay, with aggregates on the surfaces (Figure S7A,B). The external surface area measured by BET of CM-lactoglobulin increased to 57.6 m^2^/g from the base CM at 49.7 m^2^/g, suggesting protein amendments on external surfaces of the clay, while AC-lactoglobulin (β-lactoglobulin amended AC) showed both micropores and macropores on AC (Figure S7C,D). The attachment of β-lactoglobulin was validated by the FTIR spectroscopy, where the band at 1635 cm^–1^ corresponded to the β-sheets and strands in the amide I region for protein emulsions with 2% β-lactoglobulin content^85,86^ (Figure S7E). The zeta potentials for AC-lactoglobulin and CM-lactoglobulin were −36.3 ± 0.75 and −11.0 ± 1.37 mV, respectively, which were similar to their base materials. The particle size for AC-lactoglobulin and CM-lactoglobulin were 1320 ± 93 nm and 387 ± 45 nm, respectively.

### 3.5 Experimental Assays with *L. minor*

*Lemna minor* has been widely used in ecotoxicology studies with well-established toxicological testing protocols and was used as a toxicity indicator in this study. The growth of *L. minor* in blank media with CM-lactoglobulin inclusion of up to 0.2% for 7 days was increased (Figure S8). This result suggests the ability of CM-lactoglobulin to enhance growth in *L. minor*, possibly due to whey protein being a good nutrient source with nitrogen, phosphorus, and potassium for plant production.^87^ The addition of Cd, Pb, and a mixture of Cd and Pb in the media at varying concentrations exhibited significant toxicity to plant growth. This was consistent with previous findings that metals can reduce plant growth by limiting nutrient and water uptake and enhancing oxidative damage.^88^ Specifically, 0.8 ppm of Cd (Figure S9), 1.6 ppm of Pb (Figure S10), and 0.8 ppm of Cd and Pb (Figure S11) were the doses that reduced more than 50% of growth (EC_50_) in frond number, surface area, and chlorophyll content in 7 days, compared to blank media controls. Based on these findings, the same concentrations were used in detoxification studies assessing the effects of sorbent treatments.

**Figure 6:**
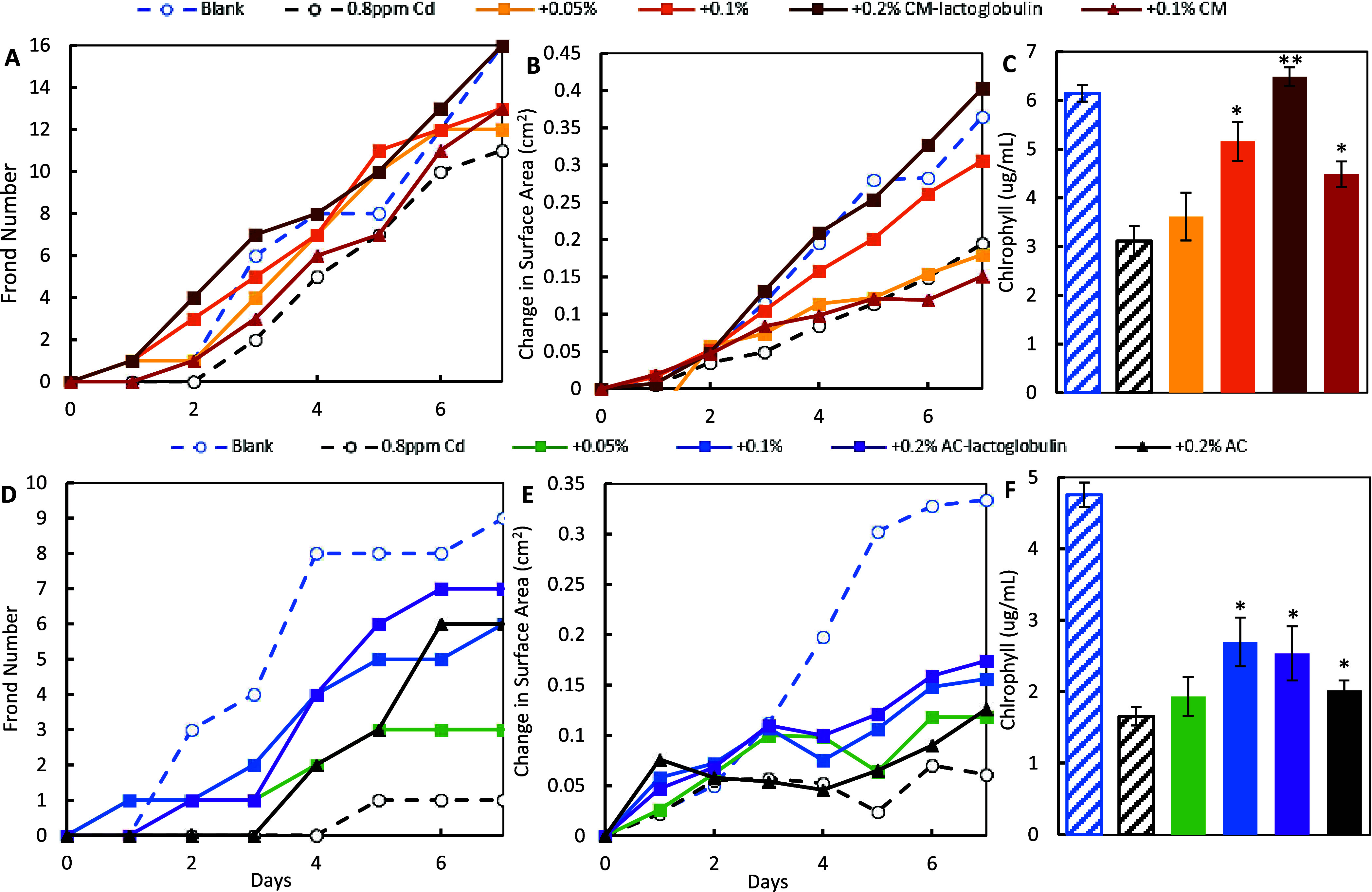
Protection of lemna from the toxicity of 0.8 ppm of Cd by 0.05, 0.1, and 0.2% CM-lactoglobulin (top panel) and AC-lactoglobulin (bottom panel) based on changes in frond number (A, D), surface area (B, E), and the chlorophyll content (C, F) (*p ≤ 0.05, **p ≤ 0.01, compared to Cd). The base CM or AC and lemna media as a blank control were included in all experiments.

**Figure 7:**
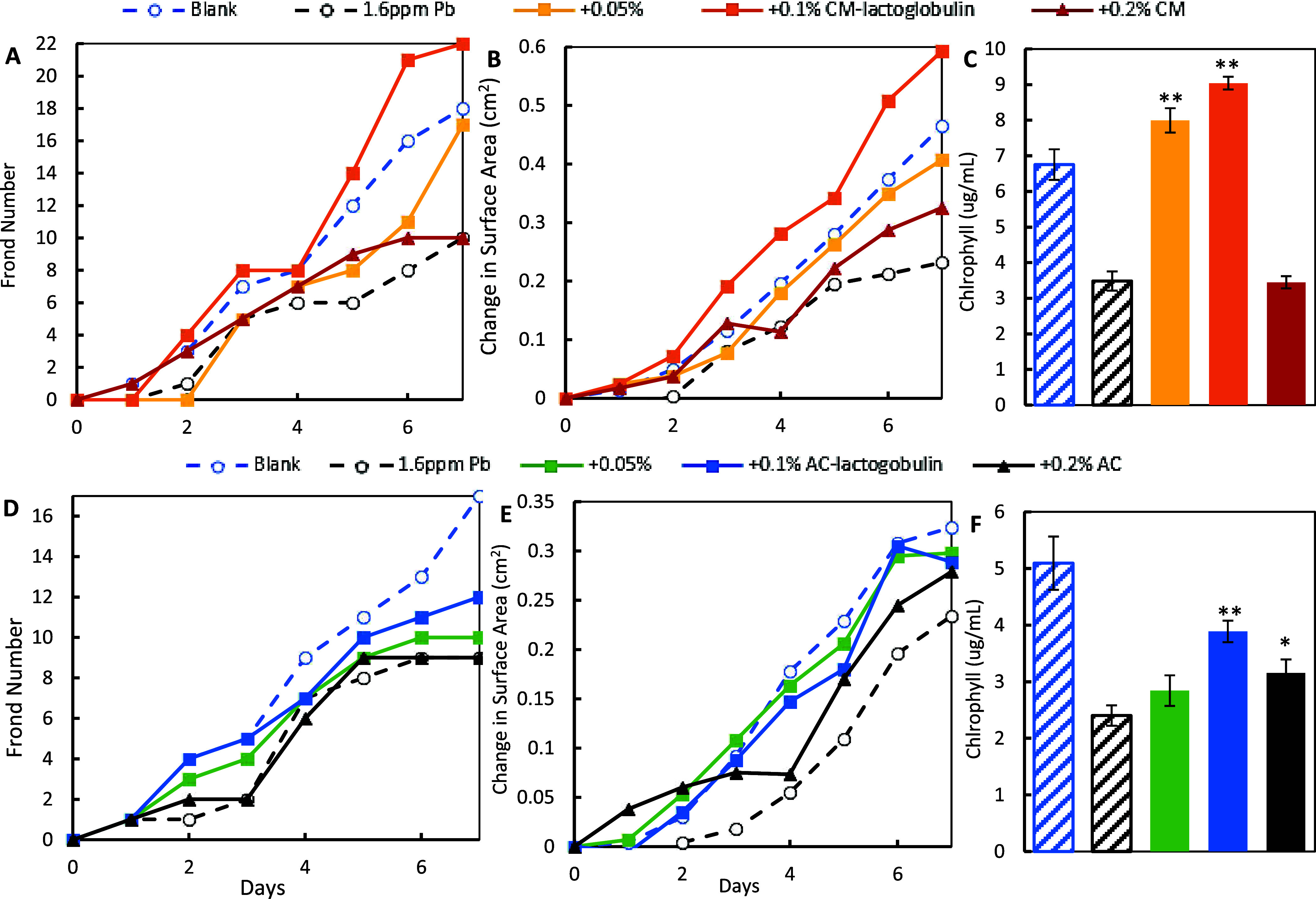
Protection of lemna from the toxicity of 1.6 ppm of Pb by 0.05 and 0.1% CM-lactoglobulin (top panel) and AC-lactoglobulin (bottom panel) based on changes in frond number (A, D), surface area (B, E), and the chlorophyll content (C, F) (*p ≤ 0.05, **p ≤ 0.01, compared to Pb). The base CM or AC and lemna media as a blank control were included in all experiments.

**Figure 8:**
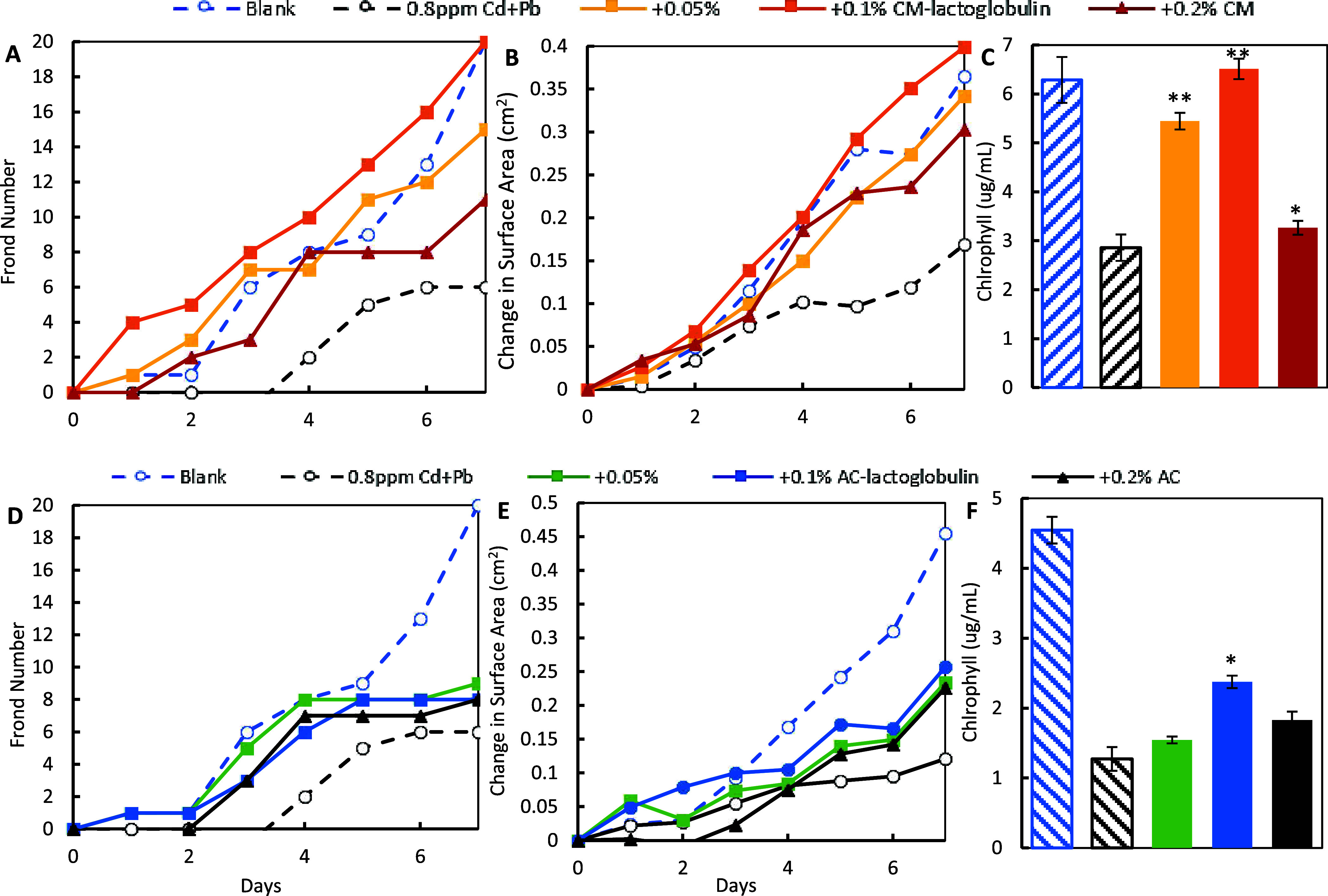
Protection of lemna from the toxicity of 0.8 ppm of Cd and Pb mixtures by 0.05 and 0.1% CM-lactoglobulin (top panel) and AC-lactoglobulin (bottom panel) based on changes in frond number (A, D), surface area (B, E), and the chlorophyll content (C, F) (*p ≤ 0.05, **p ≤ 0.01, compared to Cd and Pb). The base CM or AC and lemna media as a blank control were included in all experiments.

In the sorbent treatment study, exposure to 0.8 ppm of Cd significantly reduced lemna growth in terms of frond number, surface area, and chlorophyll content. The inclusion of CM-lactoglobulin (Figure 6A–C) and AC-lactoglobulin (Figure 6D–F) at 0.05, 0.1, and 0.2% in the media protected lemna from Cd toxicity in a dose-dependent manner. Specifically, 0.2% CM-lactoglobulin and AC-lactoglobulin most significantly increased all three growth parameters, followed by the lower doses and parent materials (CM and AC). CM-lactoglobulin showed higher protection than AC-lactoglobulin at the same dose, and 0.2% CM-lactoglobulin resulted in growth similar to that in the blank control, showing the high efficacy of CM-lactoglobulin in reacting with Cd. Similar results are shown with 1.6 ppm Pb (Figure 7) and a mixture of Cd and Pb at 0.8 ppm/metal (Figure 8), where Lemna in the 0.1% CM-lactoglobulin groups showed similar growth as in the blank controls. By comparing to the dose–response curves of heavy metals (Figures S9D, S10D, and S11D), treatment with CM lactoglobulin at 0.2 or 0.1% resulted in negligible metals remaining. AC-lactoglobulin also showed a dose-dependent protection of Lemna higher than that of AC alone.

The collective *L. minor* results showed that all of the growth parameters were highly correlative, suggesting that lemna is a sensitive model to indicate metal toxicity. Importantly, the results consistently showed that CM-lactoglobulin and AC-lactoglobulin increased the growth of lemna in a dose-dependent manner, which was higher than that of the base CM and AC alone. Their protection at low doses (up to 0.2%) suggested that the protein amendments had high efficacy in adsorbing individual metals and mixtures of metals and reducing their severe toxicity.

### 3.6 Experimental Assays with *H. vulgaris*

*Hydra vulgaris* has been widely used to indicate the toxicity of water pollutants and their sensitive responses to heavy metals have been reported.^31,89,90^ In this study, hydra morphological changes were scored from 0 to 10 to indicate toxicity. The blank media control was included in all experiments with a constant score of 10. The doses of metals that resulted in more than 50% mortality (score <5, EC_50_) at the 92 h endpoint were identified as 5 ppm of Cd, 15 ppm Pb, and 3 ppm/metal for Cd and Pb mixtures. Therefore, these concentrations were included in the detoxification study to validate the efficacy and safety of the sorbents.

**Figure 9:**
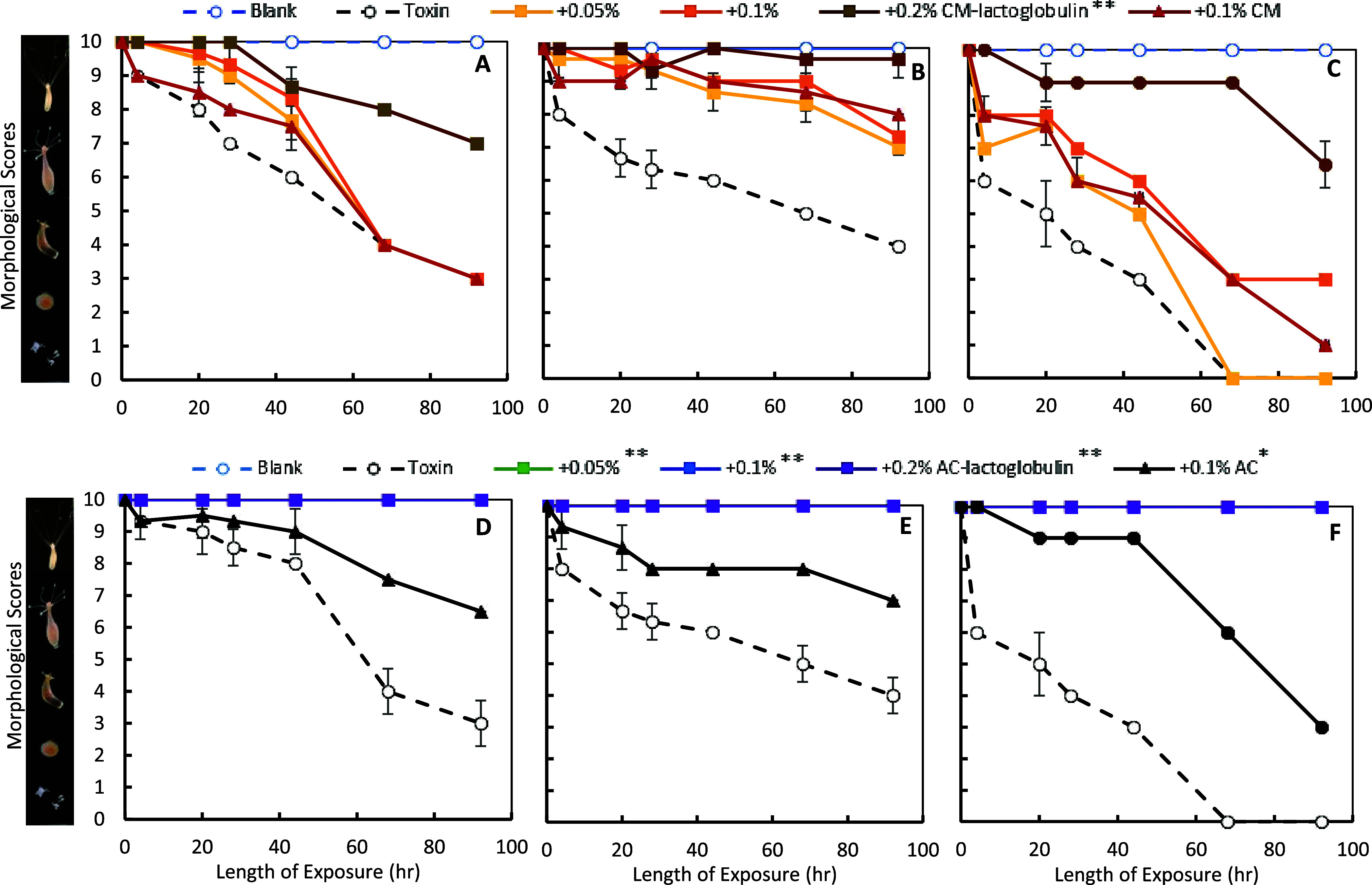
Protection of hydra from the toxicity of 5 ppm of Cd (A, D), 15 ppm of Pb (B, E), and 3 ppm of Cd and Pb (C, F) by 0.05%, 0.1%, and 0.2% CM-lactoglobulin (top panel) and AC-lactoglobulin (bottom panel). The base CM or AC and hydra media as a blank control were included in all experiments. Data represent the mean morphological score at each time point, run in triplicate (*p ≤ 0.05, **p ≤ 0.01, compared to all 3 corresponding toxin controls).

Similar to the *L. minor* results, the inclusion of CM-lactoglobulin at 0.05, 0.1, and 0.2% rates in the medium resulted in dose-dependent protection against individuals and a mixture of metals. Specifically, 0.2% CM-lactoglobulin resulted in 57, 94.5, and 65% protection from Cd, Pb, and the mixture toxicities, respectively (Figure 9A–C). This reduction in toxicity after CM-lactoglobulin treatment was higher than 0.1% of that of base CM, suggesting the interaction and binding of metals onto β-lactoglobulin amendments. The inclusion of AC-lactoglobulin at 0.05–0.2% resulted in complete protection against metal toxicities, indicating their significant binding onto AC-lactoglobulin. The complete detoxification by AC-lactoglobulin in *H. vulgaris* was more significant than in *L. minor*, possibly due to higher sensitivity to metal toxicity in the *L. minor* model. These experimental results supported the previous dosimetry study and the in silico simulation results under neutral conditions, suggesting agreement with computations.

## 4 Conclusions

In this study, we used β-lactoglobulin to enhance clay and activated carbon binding and protection properties for Cd and Pb. We initially simulated the formation of such amended materials, i.e., whether β-lactoglobulin can bind to clay and activated carbon at acidic and neutral conditions. Using docking calculations, we predicted that the protein dimer could adopt different orientations in its interaction with different materials, i.e., CM, AC20, and AC1. Subsequently, simulations were used to investigate the most energetically favorable docked poses of β-lactoglobulin protein dimer in complex with the material systems at both acidic and neutral conditions. These simulations provided insights into the complexes’ increased stability formed at acidic vs neutral conditions and the differences in binding between clay and activated carbon systems. It depicted that in all cases, the protein dimer could remain in contact with the corresponding material systems, predicting the ability of the protein to serve as an amendment for the particular materials. The simulations also investigated the binding properties of the two heavy metal ions to the material systems, which were significantly higher at neutral versus acidic conditions. Within the simulations, at neutral conditions, Cd and Pb shared comparable binding propensities in all material systems, with the former being consistently higher than the latter, and additionally, the two ions had a higher binding propensity to clay compared to activated carbon, in general. Within the simulations, we observed numerous possible binding pockets formed by the material systems, potentially contributing to the protection mechanism observed in the experiments. It is worth noting that this was valid in two different sets of simulations, examining initially server-placed ions versus randomly placed ions. This provided us with additional sampling and confirmed that different binding pockets could be possible. The binding pockets’ ability at acidic conditions was nearly eliminated because particular Asp and Glu residues involved become protonated at these conditions according to our predictions. The interaction and detoxification of metals on CM-lactoglobulin and AC-lactoglobulin were validated experimentally in *L. minor* and *H. vulgaris*, where both sorbents reduced the severe toxicity of individual metals and a mixture of Cd and Pb and consistently increased the growth parameters in a dose-dependent manner. The high efficacy of protection in the ecotoxicological models was in line with the predicted pockets for both Cd and Pb, as shown in the simulations. According to computations and experiments, the amended materials can provide augmented protection for Cd and Pb compared with parent materials. Our experimental results support agreement with computations. Notably, understanding and predicting metal ion binding sites on proteins has been the focus of extensive computational research with several challenges and underlying limitations (reviewed in^91^). Our computations were applied aiming to balance between accuracy and efficiency, and the classical MD simulations enabled us to sufficiently sample and predict potential binding pockets as well as to assess their relative stability. Additional and more in-depth mechanistic insights, e.g., on metal-protein binding energies, would require and could benefit from accurate energy calculations, including calculations performed using QM/MM, or QM/DMD,^92^ which are more suitable for methodologies investigating metal affinity. Additionally, ONIOM,^93^ a hybrid method that has been employed to study both nonmetalloproteins as well as metalloproteins, was also applied to study the interaction of Fe(II)-heme on Aβ peptide.^94^ Nevertheless, the expense of accurate energy evaluation can also severely limit the amount of sampling^92^ of the conformational space of the simulated system that could be afforded. In this study, classical force fields advantageously allowed us to provide initial insights into (i) the proteins’ interaction with the materials, combined with (ii) Pb and Cd sampling and binding onto protein-material systems at different conditions. Importantly, sampling can be considered crucial in complex systems, such as the one investigated here, involving metal ion interactions with protein-material amendments. Thus, the classical force fields used, despite their limitations in obtaining thermodynamic values,^92^ advantageously allowed the efficient exploration of different metal ion binding sites onto the protein-amended systems within conformational dynamics. We consider that the knowledge gained in this study, potentially combined with accurate state-of-the-art methods in thermodynamic calculations^92^ or with current advancements in knowledge-driven docking approaches of metal ions,^95^ can pave the way for future studies examining in detail the configuration and binding energy of a series of metals, including but not limited to Pb and Cd, to the proteins and the protein-material amended systems. This can further expand our knowledge on the potential broader applicability and functionality of the particular protein-material systems as well as on novel designed systems.

A series of studies have suggested the broad applicability of montmorillonite clays and amended montmorillonite clays in several applications due to their capacity to serve as broad-acting sorbents for a variety of toxic chemicals. The β-lactoglobulin amended materials and, particularly, clays could be potentially studied for their applicability in groundwater treatments, in the framework of clays comprising broad-acting sorbents. Additionally, “green engineered” clays amended with chlorophyll were included in both problems aiming at improving functionality toward benzene,^67^ as well as toluene and xylene for novel barrier cream formulations.^96^ Therefore, our study can provide an impetus for the future investigation of β-lactoglobulin-amended materials, such as clays, in corresponding applications.

## Supporting Information Available

Brief statement in nonsentence format of the contents of the material supplied as Supporting Information; binding pockets identified for CM-pH7 systems; binding pockets identified for AC1-pH7 systems; binding pockets identified for AC20-pH7 systems; percent occurrence of binding pockets in CM-pH7; percent occurrence of binding pockets in AC20-pH7; percent occurrence of binding pockets in AC1-pH7; lactoglobulin amendment characterized by SEM and FTIR; CM-lactoglobulin inclusion in *L. minor*; toxicity of Cd in *L. minor*; toxicity of Pb in *L. minor*; and toxicity of Cd and Pb in *L. minor* (PDF)

## References

[ref1] Aziz K. H. H.; Mustafa F. S.; Omer K. M.; Hama S.; Hamarawf R. F.; Rahman K. O. Heavy metal pollution in the aquatic environment: efficient and low-cost removal approaches to eliminate their toxicity: a review. RSC Adv. 2023, 13 (26), 17595–17610. 10.1039/D3RA00723E. 37312989 PMC10258679

[ref2] Cui X.; Wang X.; Liu B. The characteristics of heavy metal pollution in surface dust in Tangshan, a heavily industrialized city in North China, and an assessment of associated health risks. Journal of Geochemical Exploration 2020, 210, 106432 10.1016/j.gexplo.2019.106432.

[ref3] Adnan M.; Xiao B.; Xiao P.; Zhao P.; Bibi S. Heavy metal, waste, COVID-19, and rapid industrialization in this modern era—fit for sustainable future. Sustainability 2022, 14 (8), 4746 10.3390/su14084746.

[ref4] Al-Ghafari A.; Elmorsy E.; Fikry E.; Alrowaili M.; Carter W. G. The heavy metals lead and cadmium are cytotoxic to human bone osteoblasts via induction of redox stress. PLoS One 2019, 14 (11), e0225341 10.1371/journal.pone.0225341. 31756223 PMC6874340

[ref5] Humans I. W. G. O. T. E. O. C. R. T. Cadmium and cadmium compounds. In Beryllium, Cadmium, Mercury, and Exposures in the Glass Manufacturing Industry; International Agency for Research on Cancer, 1993.

[ref6] Schaefer H. R.; Flannery B. M.; Crosby L.; Jones-Dominic O. E.; Punzalan C.; Middleton K. A systematic review of adverse health effects associated with oral cadmium exposure. Regul. Toxicol. Pharmacol. 2022, 134, 105243 10.1016/j.yrtph.2022.105243. 35981600

[ref7] Wang M.; Chen Z.; Song W.; Hong D.; Huang L.; Li Y. A review on cadmium exposure in the population and intervention strategies against cadmium toxicity. Bull. Environ. Contam. Toxicol. 2021, 106, 65–74. 10.1007/s00128-020-03088-1. 33486543

[ref8] Jean J.; Sirot V.; Hulin M.; Le Calvez E.; Zinck J.; Noël L.; Vasseur P.; Nesslany F.; Gorecki S.; Guérin T. Dietary exposure to cadmium and health risk assessment in children–Results of the French infant total diet study. Food Chem. Toxicol. 2018, 115, 358–364. 10.1016/j.fct.2018.03.031. 29580822

[ref9] Huff J.; Lunn R. M.; Waalkes M. P.; Tomatis L.; Infante P. F. Cadmium-induced cancers in animals and in humans. International journal of occupational and environmental health 2007, 13 (2), 202–212. 10.1179/oeh.2007.13.2.202. 17718178 PMC3399253

[ref10] Zhang S.; Cui M.; Chen J.; Ding Z.; Wang X.; Mu Y.; Meng C. Modification of synthetic zeolite X by thiourea and its adsorption for Cd (II). Mater. Lett. 2019, 236, 233–235. 10.1016/j.matlet.2018.10.100.

[ref11] Kumar A.; Kumar A.; MMS C.-P; Chaturvedi A. K.; Shabnam A. A.; Subrahmanyam G.; Mondal R.; Gupta D. K.; Malyan S. K.; Kumar S. S. Lead toxicity: health hazards, influence on food chain, and sustainable remediation approaches. Int. J. Environ. Res. Public Health 2020, 17 (7), 2179 10.3390/ijerph17072179. 32218253 PMC7177270

[ref12] Flora G.; Gupta D.; Tiwari A. Toxicity of lead: a review with recent updates. Interdisciplinary toxicology 2012, 5 (2), 47–58. 10.2478/v10102-012-0009-2. 23118587 PMC3485653

[ref13] Boskabady M.; Marefati N.; Farkhondeh T.; Shakeri F.; Farshbaf A.; Boskabady M. H. The effect of environmental lead exposure on human health and the contribution of inflammatory mechanisms, a review. Environ. Int. 2018, 120, 404–420. 10.1016/j.envint.2018.08.013. 30125858

[ref14] Matlock M. M.; Howerton B. S.; Atwood D. A. Chemical precipitation of heavy metals from acid mine drainage. Water research 2002, 36 (19), 4757–4764. 10.1016/S0043-1354(02)00149-5. 12448518

[ref15] Wang Q.; Yu J.; Chen X.; Du D.; Wu R.; Qu G.; Guo X.; Jia H.; Wang T. Non-thermal plasma oxidation of Cu (II)-EDTA and simultaneous Cu (II) elimination by chemical precipitation. Journal of environmental management 2019, 248, 109237 10.1016/j.jenvman.2019.07.008. 31310932

[ref16] Sun Y.; Zhou S.; Pan S.-Y.; Zhu S.; Yu Y.; Zheng H. Performance evaluation and optimization of flocculation process for removing heavy metal. Chemical Engineering Journal 2020, 385, 123911 10.1016/j.cej.2019.123911.

[ref17] Zhang Q.; Ye X.; Li H.; Chen D.; Xiao W.; Zhao S.; Xiong R.; Li J. Cumulative effects of pyrolysis temperature and process on properties, chemical speciation, and environmental risks of heavy metals in magnetic biochar derived from coagulation-flocculation sludge of swine wastewater. Journal of Environmental Chemical Engineering 2020, 8 (6), 104472 10.1016/j.jece.2020.104472.

[ref18] Bashir A.; Malik L. A.; Ahad S.; Manzoor T.; Bhat M. A.; Dar G.; Pandith A. H. Removal of heavy metal ions from aqueous system by ion-exchange and biosorption methods. Environmental Chemistry Letters 2019, 17, 729–754. 10.1007/s10311-018-00828-y.

[ref19] Hoseinian F. S.; Rezai B.; Kowsari E.; Safari M. A hybrid neural network/genetic algorithm to predict Zn (II) removal by ion flotation. Sep. Sci. Technol. 2020, 55 (6), 1197–1206. 10.1080/01496395.2019.1582543.

[ref20] Saleem H.; Pal P.; Haija M. A.; Banat F. Regeneration and reuse of bio-surfactant to produce colloidal gas aphrons for heavy metal ions removal using single and multistage cascade flotation. Journal of Cleaner Production 2019, 217, 493–502. 10.1016/j.jclepro.2019.01.216.

[ref21] Shrestha R.; Ban S.; Devkota S.; Sharma S.; Joshi R.; Tiwari A. P.; Kim H. Y.; Joshi M. K. Technological trends in heavy metals removal from industrial wastewater: A review. Journal of Environmental Chemical Engineering 2021, 9 (4), 105688 10.1016/j.jece.2021.105688.

[ref22] Qasem N. A.; Mohammed R. H.; Lawal D. U. Removal of heavy metal ions from wastewater: A comprehensive and critical review. npj Clean Water 2021, 4 (1), 36 10.1038/s41545-021-00127-0.

[ref23] Gupta V. K.; Carrott P.; Ribeiro Carrott M.; Suhas Low-cost adsorbents: growing approach to wastewater treatment—a review. Critical reviews in environmental science and technology 2009, 39 (10), 783–842. 10.1080/10643380801977610.

[ref24] Alengebawy A.; Abdelkhalek S. T.; Qureshi S. R.; Wang M.-Q. Heavy metals and pesticides toxicity in agricultural soil and plants: Ecological risks and human health implications. Toxics 2021, 9 (3), 42 10.3390/toxics9030042. 33668829 PMC7996329

[ref25] Vitali M.; Antonucci A.; Owczarek M.; Guidotti M.; Astolfi M. L.; Manigrasso M.; Avino P.; Bhattacharya B.; Protano C. Air quality assessment in different environmental scenarios by the determination of typical heavy metals and Persistent Organic Pollutants in native lichen Xanthoria parietina. Environ. Pollut. 2019, 254, 113013 10.1016/j.envpol.2019.113013. 31415978

[ref26] Colman Lerner J. E.; Elordi M. L.; Orte M. A.; Giuliani D.; De Los Angeles Gutierrez M.; Sanchez E.; Sambeth J. E.; Porta A. A. Exposure and risk analysis to particulate matter, metals, and polycyclic aromatic hydrocarbon at different workplaces in Argentina. Environmental Science and Pollution Research 2018, 25, 8487–8496. 10.1007/s11356-017-1101-0. 29308573

[ref27] El-Mufleh A.; Béchet B.; Basile-Doelsch I.; Geffroy-Rodier C.; Gaudin A.; Ruban V. Distribution of PAHs and trace metals in urban stormwater sediments: combination of density fractionation, mineralogy and microanalysis. Environmental Science and Pollution Research 2014, 21, 9764–9776. 10.1007/s11356-014-2850-7. 24764003

[ref28] Lv D.; Zhu T.; Liu R.; Li X.; Zhao Y.; Sun Y.; Wang H.; Zhang F.; Zhao Q. Effects of co-processing sewage sludge in the cement kiln on PAHs, heavy metals emissions and the surrounding environment. International Journal of Environmental Research and Public Health 2018, 15 (4), 698 10.3390/ijerph15040698. 29642474 PMC5923740

[ref29] Wang M.; Orr A. A.; Jakubowski J. M.; Bird K. E.; Casey C. M.; Hearon S. E.; Tamamis P.; Phillips T. D. Enhanced adsorption of per-and polyfluoroalkyl substances (PFAS) by edible, nutrient-amended montmorillonite clays. Water research 2021, 188, 116534 10.1016/j.watres.2020.116534. 33125992 PMC7725962

[ref30] Hearon S. E.; Wang M.; Phillips T. D. Strong adsorption of dieldrin by parent and processed montmorillonite clays. Environmental toxicology and chemistry 2020, 39 (3), 517–525. 10.1002/etc.4642. 31756776 PMC7047628

[ref31] Wang M.; Bera G.; Mitra K.; Wade T. L.; Knap A. H.; Phillips T. D. Tight sorption of arsenic, cadmium, mercury, and lead by edible activated carbon and acid-processed montmorillonite clay. Environmental Science and Pollution Research 2021, 28, 6758–6770. 10.1007/s11356-020-10973-z. 33009611 PMC7855320

[ref32] Phillips T. D.; Wang M.; Elmore S. E.; Hearon S.; Wang J.-S. NovaSil clay for the protection of humans and animals from aflatoxins and other contaminants. Clays and clay minerals 2019, 67 (1), 99–110. 10.1007/s42860-019-0008-x. 32943795 PMC7494129

[ref33] Mitchell N. J.; Kumi J.; Aleser M.; Elmore S. E.; Rychlik K. A.; Zychowski K. E.; Romoser A. A.; Phillips T. D.; Ankrah N.-A. Short-term safety and efficacy of calcium montmorillonite clay (UPSN) in children. American journal of tropical medicine and hygiene 2014, 91 (4), 777 10.4269/ajtmh.14-0093. 25135766 PMC4183404

[ref34] Wang M.; Orr A. A.; He S.; Dalaijamts C.; Chiu W. A.; Tamamis P.; Phillips T. D. Montmorillonites can tightly bind glyphosate and paraquat reducing toxin exposures and toxicity. ACS omega 2019, 4 (18), 17702–17713. 10.1021/acsomega.9b02051. 31681876 PMC6822125

[ref35] Maki C.; Haney S.; Wang M.; Ward S.; Rude B.; Bailey R.; Harvey R.; Phillips T. Calcium montmorillonite clay for the reduction of aflatoxin residues in milk and dairy products. J. Dairy Vet. Sci. 2017, 2 (3), 1–8. 10.19080/JDVS.2017.02.555587.

[ref36] Pollock B. H.; Elmore S.; Romoser A.; Tang L.; Kang M.-s.; Xue K.; Rodriguez M.; Dierschke N. A.; Hayes H. G.; Hansen H. A. Intervention trial with calcium montmorillonite clay in a south Texas population exposed to aflatoxin. Food Addit. Contam.: Part A 2016, 33 (8), 1346–1354. 10.1080/19440049.2016.1198498. PMC5145309 27321368

[ref37] Tongur T.; Ayranci E. Adsorption and electrosorption of paraquat, diquat and difenzoquat from aqueous solutions onto activated carbon cloth as monitored by in-situ uv–visible spectroscopy. Journal of Environmental Chemical Engineering 2021, 9 (4), 105566 10.1016/j.jece.2021.105566.

[ref38] Millward R. N.; Bridges T. S.; Ghosh U.; Zimmerman J. R.; Luthy R. G. Addition of activated carbon to sediments to reduce PCB bioaccumulation by a polychaete (Neanthes arenaceodentata) and an amphipod (Leptocheirus plumulosus). Environ. Sci. Technol. 2005, 39 (8), 2880–2887. 10.1021/es048768x. 15884389

[ref39] Gómez-Serrano V.; Adame-Pereira M.; Alexandre-Franco M.; Fernández-González C. Adsorption of bisphenol A by activated carbon developed from PET waste by KOH activation. Environmental Science and Pollution Research 2021, 28, 24342–24354. 10.1007/s11356-020-08428-6. 32212082

[ref40] Rodzik A.; Pomastowski P.; Sagandykova G. N.; Buszewski B. Interactions of whey proteins with metal ions. International journal of molecular sciences 2020, 21 (6), 2156 10.3390/ijms21062156. 32245108 PMC7139725

[ref41] Le Maux S.; Bouhallab S.; Giblin L.; Brodkorb A.; Croguennec T. Bovine β-lactoglobulin/fatty acid complexes: binding, structural, and biological properties. Dairy science & technology 2014, 94, 409–426. 10.1007/s13594-014-0160-y. 25110551 PMC4121524

[ref42] Peydayesh M.; Bolisetty S.; Mohammadi T.; Mezzenga R. Assessing the binding performance of amyloid–carbon membranes toward heavy metal ions. Langmuir 2019, 35 (11), 4161–4170. 10.1021/acs.langmuir.8b04234. 30811203

[ref43] Cho Y.; Singh H.; Creamer L. K. Heat-induced interactions of β-lactoglobulin A and κ-casein B in a model system. Journal of Dairy Research 2003, 70 (1), 61–71. 10.1017/S0022029902005642. 12617394

[ref44] Bolisetty S.; Mezzenga R. Amyloid–carbon hybrid membranes for universal water purification. Nature Nanotechnol. 2016, 11 (4), 365–371. 10.1038/nnano.2015.310. 26809058

[ref45] Fan Y.; Lan H.; Qi Z.; Liu R.; Hu C. Removal of nickel and copper ions in strongly acidic conditions by in-situ formed amyloid fibrils. Chemosphere 2022, 297, 134241 10.1016/j.chemosphere.2022.134241. 35259361

[ref46] Wang M.; Hearon S. E.; Johnson N. M.; Phillips T. D. Development of broad-acting clays for the tight adsorption of benzo [a] pyrene and aldicarb. Appl. Clay Sci. 2019, 168, 196–202. 10.1016/j.clay.2018.11.010. 31435120 PMC6703832

[ref47] Rivenbark K. J.; Wang M.; Lilly K.; Tamamis P.; Phillips T. D. Development and characterization of chlorophyll-amended montmorillonite clays for the adsorption and detoxification of benzene. Water Res. 2022, 221, 118788 10.1016/j.watres.2022.118788. 35777320 PMC9662585

[ref48] Hearon S. E.; Orr A. A.; Moyer H.; Wang M.; Tamamis P.; Phillips T. D. Montmorillonite clay-based sorbents decrease the bioavailability of per-and polyfluoroalkyl substances (PFAS) from soil and their translocation to plants. Environmental Research 2022, 205, 112433 10.1016/j.envres.2021.112433. 34875259 PMC8760172

[ref49] Orr A. A.; He S.; Wang M.; Goodall A.; Hearon S. E.; Phillips T. D.; Tamamis P. Insights into the interactions of bisphenol and phthalate compounds with unamended and carnitine-amended montmorillonite clays. Computers & chemical engineering 2020, 143, 107063 10.1016/j.compchemeng.2020.107063. 33122868 PMC7591107

[ref50] Fashina B.; Deng Y.; Cagin T.; Cygan R. Insights on adsorption of pyocyanin in montmorillonite using molecular dynamics simulation. Phys. Chem. Chem. Phys. 2024, 26 (13), 10310–10322. 10.1039/D3CP05762C. 38498351

[ref51] Liu Y.; Zhang D.; Ren B.; Gong X.; Xu L.; Feng Z.-Q.; Chang Y.; He Y.; Zheng J. Molecular simulations and understanding of antifouling zwitterionic polymer brushes. J. Mater. Chem. B 2020, 8 (17), 3814–3828. 10.1039/D0TB00520G. 32227061

[ref52] Yeates T. O.; McPherson A. The structure of bovine β-lactoglobulin in crystals grown at pH 3.8 exhibiting novel threefold twinning. Acta Crystallographica Section F: Structural Biology. Communications 2019, 75 (10), 640–645. 10.1107/S2053230X1901224X. PMC6777136 31584012

[ref53] Loch J. I.; Bonarek P.; Tworzydło M.; Polit A.; Hawro B.; Łach A.; Ludwin E.; Lewiński K. Engineered β-lactoglobulin produced in E. coli: purification, biophysical and structural characterisation. Molecular Biotechnology 2016, 58, 605–618. 10.1007/s12033-016-9960-z. 27380951 PMC5035327

[ref54] Kaplan W.; Littlejohn T. G. Swiss-PDB viewer (deep view). Briefings in bioinformatics 2001, 2 (2), 195–197. 10.1093/bib/2.2.195. 11465736

[ref55] Ismer J.; Rose A. S.; Tiemann J. K.; Goede A.; Preissner R.; Hildebrand P. W. SL2: an interactive webtool for modeling of missing segments in proteins. Nucleic acids research 2016, 44 (W1), W390–W394. 10.1093/nar/gkw297. 27105847 PMC4987885

[ref56] Rostkowski M.; Olsson M. H.; So̷ndergaard C. R.; Jensen J. H. Graphical analysis of pH-dependent properties of proteins predicted using PROPKA. BMC Struct. Biol. 2011, 11, 6 10.1186/1472-6807-11-6. 21269479 PMC3038139

[ref57] Qin B. Y.; Bewley M. C.; Creamer L. K.; Baker H. M.; Baker E. N.; Jameson G. B. Structural basis of the Tanford transition of bovine β-lactoglobulin. Biochemistry 1998, 37 (40), 14014–14023. 10.1021/bi981016t. 9760236

[ref58] Huang J.; MacKerell A. D. Jr CHARMM36 all-atom additive protein force field: Validation based on comparison to NMR data. Journal of computational chemistry 2013, 34 (25), 2135–2145. 10.1002/jcc.23354. 23832629 PMC3800559

[ref59] Orr A. A.; Wang M.; Beykal B.; Ganesh H. S.; Hearon S. E.; Pistikopoulos E. N.; Phillips T. D.; Tamamis P. Combining experimental isotherms, minimalistic simulations, and a model to understand and predict chemical adsorption onto montmorillonite clays. ACS omega 2021, 6 (22), 14090–14103. 10.1021/acsomega.1c00481. 34124432 PMC8190805

[ref60] Jo S.; Kim T.; Iyer V. G.; Im W. CHARMM-GUI: a web-based graphical user interface for CHARMM. Journal of computational chemistry 2008, 29 (11), 1859–1865. 10.1002/jcc.20945. 18351591

[ref61] Choi Y. K.; Kern N. R.; Kim S.; Kanhaiya K.; Afshar Y.; Jeon S. H.; Jo S.; Brooks B. R.; Lee J.; Tadmor E. B.; et al. CHARMM-GUI Nanomaterial Modeler for Modeling and Simulation of Nanomaterial Systems. J. Chem. Theory Comput 2022, 18 (1), 479–493. 10.1021/acs.jctc.1c00996. 34871001 PMC8752518

[ref62] Heinz H.; Lin T.-J.; Kishore Mishra R.; Emami F. S. Thermodynamically consistent force fields for the assembly of inorganic, organic, and biological nanostructures: the INTERFACE force field. Langmuir 2013, 29 (6), 1754–1765. 10.1021/la3038846. 23276161

[ref63] Brooks B. R.; Brooks C. L. III; Mackerell A. D. Jr; Nilsson L.; Petrella R. J.; Roux B.; Won Y.; Archontis G.; Bartels C.; Boresch S. CHARMM: the biomolecular simulation program. J. Comput. Chem. 2009, 30 (10), 1545–1614. 10.1002/jcc.21287. 19444816 PMC2810661

[ref64] Lee J.; Cheng X.; Jo S.; MacKerell A. D.; Klauda J. B.; Im W. CHARMM-GUI input generator for NAMD, GROMACS, AMBER, OpenMM, and CHARMM/OpenMM simulations using the CHARMM36 additive force field. Biophysical journal 2016, 110 (3), 641a 10.1016/j.bpj.2015.11.3431. PMC4712441 26631602

[ref65] Harris P. J.; Liu Z.; Suenaga K. Imaging the atomic structure of activated carbon. J. Phys.: Condens. Matter 2008, 20 (36), 362201 10.1088/0953-8984/20/36/362201.

[ref66] Yang P.-Y.; Ju S.-P.; Huang S.-M. Predicted structural and mechanical properties of activated carbon by molecular simulation. Comput. Mater. Sci. 2018, 143, 43–54. 10.1016/j.commatsci.2017.10.051.

[ref67] Rivenbark K. J.; Lilly K.; Wang M.; Tamamis P.; Phillips T. D. Green-engineered clay-and carbon-based composite materials for the adsorption of benzene from air. Journal of Environmental. Chemical Engineering 2024, 12, 111836 10.1016/j.jece.2023.111836. PMC10993424 38576544

[ref68] Vanommeslaeghe K.; Hatcher E.; Acharya C.; Kundu S.; Zhong S.; Shim J.; Darian E.; Guvench O.; Lopes P.; Vorobyov I. CHARMM general force field: A force field for drug-like molecules compatible with the CHARMM all-atom additive biological force fields. J. Comput. Chem. 2010, 31 (4), 671–690. 10.1002/jcc.21367. 19575467 PMC2888302

[ref69] Ogawa-Fuse C.; Morisaki N.; Shima K.; Hotta M.; Sugata K.; Ichihashi T.; Oguri M.; Yoshida O.; Fujimura T. Impact of water exposure on skin barrier permeability and ultrastructure. Contact Dermatitis 2019, 80 (4), 228–233. 10.1111/cod.13174. 30417381

[ref70] Chen P.; Ke Y.; Lu Y.; Du Y.; Li J.; Yan H.; Zhao H.; Zhou Y.; Yang Y. DLIGAND2: an improved knowledge-based energy function for protein–ligand interactions using the distance-scaled, finite, ideal-gas reference state. J. Cheminf. 2019, 11 (1), 1–11. 10.1186/s13321-019-0373-4. PMC6686496 31392430

[ref71] Lu C.-H.; Chen C.-C.; Yu C.-S.; Liu Y.-Y.; Liu J.-J.; Wei S.-T.; Lin Y.-F. MIB2: metal ion-binding site prediction and modeling server. Bioinformatics 2022, 38 (18), 4428–4429. 10.1093/bioinformatics/btac534. 35904542

[ref72] Won Y. Force field for monovalent, divalent, and trivalent cations developed under the solvent boundary potential. J. Phys. Chem. A 2012, 116 (47), 11763–11767. 10.1021/jp309150r. 23102428

[ref73] Eastman P.; Swails J.; Chodera J. D.; McGibbon R. T.; Zhao Y.; Beauchamp K. A.; Wang L.-P.; Simmonett A. C.; Harrigan M. P.; Stern C. D. OpenMM 7: Rapid development of high performance algorithms for molecular dynamics. PLoS Comput. Biol. 2017, 13 (7), e1005659 10.1371/journal.pcbi.1005659. 28746339 PMC5549999

[ref74] Wang M.; Lilly K.; Martin L. M.; Xu W.; Tamamis P.; Phillips T. D. Adsorption and removal of polystyrene nanoplastics from water by green-engineered clays. Water Res. 2024, 249, 120944 10.1016/j.watres.2023.120944. 38070346 PMC11824905

[ref75] Humphrey W.; Dalke A.; Schulten K. VMD: visual molecular dynamics. J. Mol. Graphics 1996, 14 (1), 33–38. 10.1016/0263-7855(96)00018-5. 8744570

[ref76] Wang M.; Rivenbark K. J.; Nikkhah H.; Beykal B.; Phillips T. D. In vitro and in vivo remediation of per-and polyfluoroalkyl substances by processed and amended clays and activated carbon in soil. Applied Soil Ecology 2024, 196, 105285 10.1016/j.apsoil.2024.105285. 38463139 PMC10919550

[ref77] Wang M.; Rivenbark K.; Gong J.; Wright F. A.; Phillips T. D. Application of edible montmorillonite clays for the adsorption and detoxification of microcystin. ACS applied bio materials 2021, 4 (9), 7254–7265. 10.1021/acsabm.1c00779. PMC8570584 34746680

[ref78] Bondos S. E.; Bicknell A. Detection and prevention of protein aggregation before, during, and after purification. Analytical biochemistry 2003, 316 (2), 223–231. 10.1016/S0003-2697(03)00059-9. 12711344

[ref79] Wang M.; Maki C. R.; Deng Y.; Tian Y.; Phillips T. D. Development of high capacity enterosorbents for aflatoxin B1 and other hazardous chemicals. Chemical research in toxicology 2017, 30 (9), 1694–1701. 10.1021/acs.chemrestox.7b00154. 28768106 PMC6684212

[ref80] Rice P. A.; Yang S.-W.; Mizuuchi K.; Nash H. A. Crystal structure of an IHF-DNA complex: a protein-induced DNA U-turn. Cell 1996, 87 (7), 1295–1306. 10.1016/S0092-8674(00)81824-3. 8980235

[ref81] Fredslund F.; Laursen N. S.; Roversi P.; Jenner L.; Oliveira C. L.; Pedersen J. S.; Nunn M. A.; Lea S. M.; Discipio R.; Sottrup-Jensen L. Structure of and influence of a tick complement inhibitor on human complement component 5. Nat. Immunol. 2008, 9 (7), 753–760. 10.1038/ni.1625. 18536718

[ref82] Ökvist M.; Dey R.; Sasso S.; Grahn E.; Kast P.; Krengel U. 1.6 Å crystal structure of the secreted chorismate mutase from Mycobacterium tuberculosis: novel fold topology revealed. Journal of molecular biology 2006, 357 (5), 1483–1499. 10.1016/j.jmb.2006.01.069. 16499927

[ref83] 20079, I. D. Water quality – determination of the toxic effect of water constituents and waste water to duckweed (Lemna minor) – Duckweed growth inhibition test, 2004.

[ref84] Balasco N.; Ferraro G.; Loreto D.; Iacobucci I.; Monti M.; Merlino A. Cisplatin binding to β-lactoglobulin: A structural study. Dalton Transactions 2020, 49 (35), 12450–12457. 10.1039/D0DT02582H. 32852026

[ref85] Eissa A. S.; Puhl C.; Kadla J. F.; Khan S. A. Enzymatic cross-linking of β-lactoglobulin: Conformational properties using FTIR spectroscopy. Biomacromolecules 2006, 7 (6), 1707–1713. 10.1021/bm050928p. 16768388

[ref86] Schestkowa H.; Drusch S.; Wagemans A. M. FTIR analysis of β-lactoglobulin at the oil/water-interface. Food chemistry 2020, 302, 125349 10.1016/j.foodchem.2019.125349. 31442700

[ref87] Ayşen A.; Durmus S. The effects of whey application on the soil biological properties and plant growth. Eurasian J. Soil Sci. 2020, 9 (4), 349–355. 10.18393/ejss.785380.

[ref88] Ali B.; Gill R. A. Heavy metal toxicity in plants: Recent insights on physiological and molecular aspects, volume II. Front. Plant Sci. 2022, 13, 1016257 10.3389/fpls.2022.1016257. 36340414 PMC9634536

[ref89] McKinley K.; McLellan I.; Gagné F.; Quinn B. The toxicity of potentially toxic elements (Cu, Fe, Mn, Zn and Ni) to the cnidarian Hydra attenuata at environmentally relevant concentrations. Science of the total environment 2019, 665, 848–854. 10.1016/j.scitotenv.2019.02.193. 30790757

[ref90] Holdway D. A.; Lok K.; Semaan M. The acute and chronic toxicity of cadmium and zinc to two hydra species. Environmental toxicology 2001, 16 (6), 557–565. 10.1002/tox.10017. 11769255

[ref91] Ye N.; Zhou F.; Liang X.; Chai H.; Fan J.; Li B.; Zhang J. A Comprehensive Review of Computation-Based Metal-Binding Prediction Approaches at the Residue Level. BioMed Res. Int. 2022, 2022 (1), 8965712 10.1155/2022/8965712. 35402609 PMC8989566

[ref92] Reilley D. J.; Hennefarth M. R.; Alexandrova A. N. The case for enzymatic competitive metal affinity methods. ACS catalysis 2020, 10 (3), 2298–2307. 10.1021/acscatal.9b04831. 34012720 PMC8130888

[ref93] Chung L. W.; Sameera W.; Ramozzi R.; Page A. J.; Hatanaka M.; Petrova G. P.; Harris T. V.; Li X.; Ke Z.; Liu F. The ONIOM method and its applications. Chem. Rev. 2015, 115 (12), 5678–5796. 10.1021/cr5004419. 25853797

[ref94] Azimi S.; Rauk A. The Binding of Fe (II)–Heme to the Amyloid Beta Peptide of Alzheimer’s Disease: QM/MM Investigations. J. Chem. Theory Comput. 2012, 8 (12), 5150–5158. 10.1021/ct300716p. 26593204

[ref95] Clemente C. M.; Prieto J. M.; Martí M. Unlocking Precision Docking for Metalloproteins. J. Chem. Inf. Model. 2024, 64, 1581 10.1021/acs.jcim.3c01853. 38373276

[ref96] Wang M.; Phillips T. D. Green-engineered barrier creams with montmorillonite-chlorophyll clays as adsorbents for benzene, toluene, and xylene. Separations 2023, 10 (4), 237 10.3390/separations10040237. 37251084 PMC10214870

