# Supplementary material for: β-Lactoglobulin Enhances Clay and Activated Carbon Binding and Protection Properties for Cadmium and Lead
Source: Ind Eng Chem Res. 2024 Sep 6;63(37):16124–40. doi: 10.1021/acs.iecr.4c01774 (PMC11417999; doi:10.1021/acs.iecr.4c01774)
Supplement: Supplementary file 1 — ie4c01774_si_001.pdf [file ie4c01774_si_001.pdf]

β-lactoglobulin enhances clay and activated carbon binding and protection properties for cadmium and lead

Supporting Information

Kendall Lilly[a], Meichen Wang[c,d], Asuka Orr[b], Sarah E. Bondos[e], Timothy D. Phillips[c,d,*], and Phanourios Tamamis[a,b,*]

[a]Department of Materials Science and Engineering, College of Engineering, Texas A&M University, College Station, TX 77843, USA

[b]Artie McFerrin Department of Chemical Engineering, College of Engineering, Texas A&M University, College Station, TX 77843, USA

[c]Department of Veterinary Physiology and Pharmacology, College of Veterinary Medicine and Biomedical Sciences, Texas A&M University, College Station, TX 77843, USA

[d]Interdisciplinary Faculty of Toxicology, College of Veterinary Medicine and Biomedical Sciences, Texas A&M University, College Station, TX 77843, USA

[e]Department of Molecular and Cellular Medicine, Texas A&M Health Science Center, Texas A&M University, College Station, TX 77843, USA

*Corresponding authors:

Phanourios Tamamis: tamamis@tamu.edu

Timothy D. Phillips: tphillips@cvm.tamu.edu

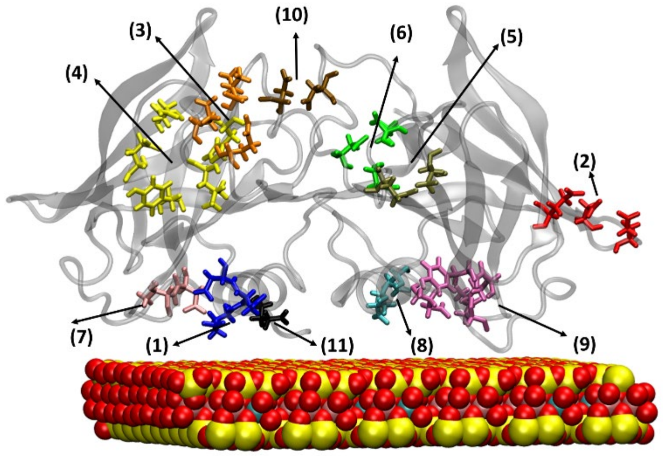

Figure S1: Zoomed out image of the binding pockets identified for CM-pH7 systems. The protein dimer is shown in transparent new cartoon representation, CM is shown in vdw representation, and the identified binding pockets are shown in various colors of licorice representation. Binding pockets are numbered, corresponding to their number in **Table 2**.

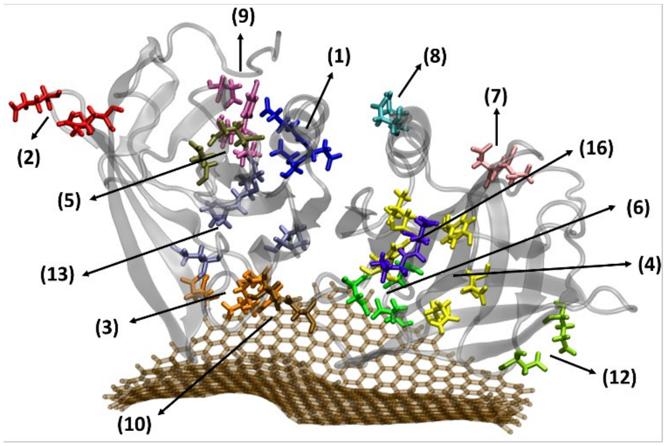

Figure S2: Zoomed out image of the binding pockets identified for AC1-pH7 systems. The protein dimer is shown in transparent new cartoon representation, CM is shown in vdw representation, and the identified binding pockets are shown in various colors of licorice representation. Binding pockets are numbered, corresponding to their number in **Table 2**.

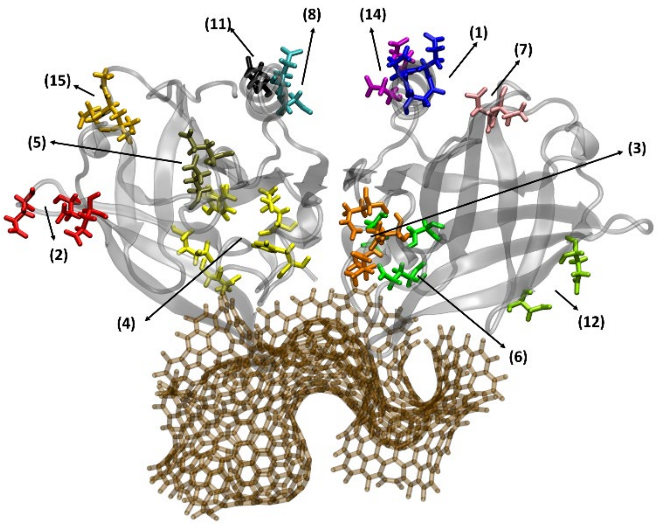

Figure S3: Zoomed out image of the binding pockets identified for AC20-pH7 systems. The protein dimer is shown in transparent new cartoon representation, CM is shown in vdw representation, and the identified binding pockets are shown in various colors of licorice representation. Binding pockets are numbered, corresponding to their number in **Table 2**.

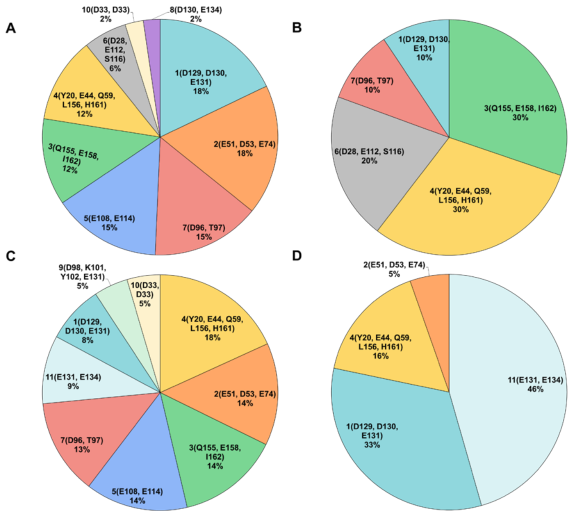

Figure S4: The percent occurrence of binding pockets in the material system CM-pH7 broken down by A) server-placed Cd ions, B) randomly-placed Cd ions, C) server-placed Pb ions, and D) randomly-placed docked Pb ions.

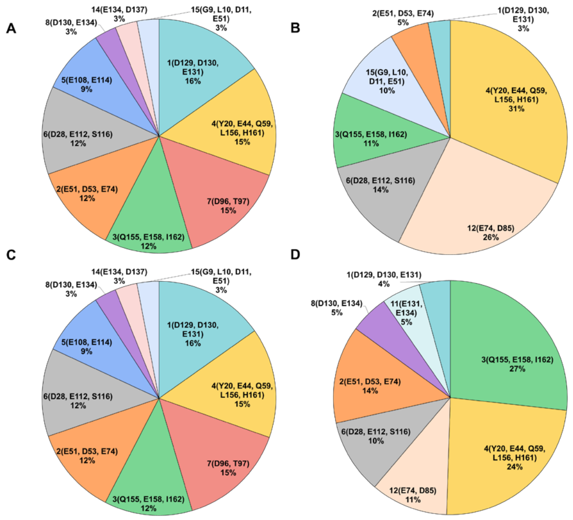

Figure S5: The percent occurrence of binding pockets in the material system AC20-pH7 broken down by A) server-placed Cd ions, B) randomly-placed Cd ions, C) server-placed Pb ions, and D) randomly-placed docked Pb ions.

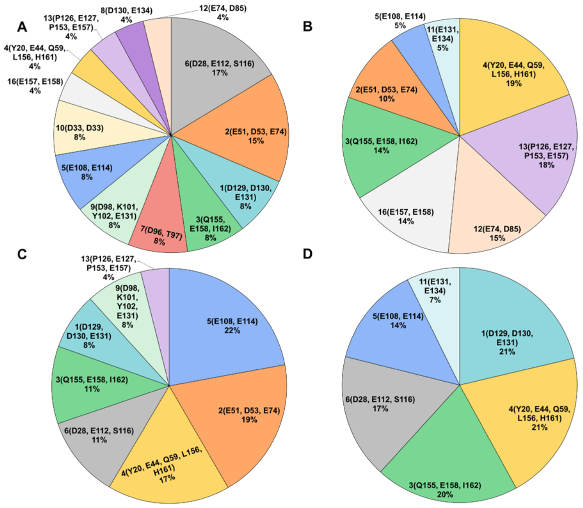

Figure S6: The percent occurrence of binding pockets in the material system AC1-pH7 broken down by A) server-placed Cd ions, B) randomly-placed Cd ions, C) server-placed Pb ions, and D) randomly-placed docked Pb ions.

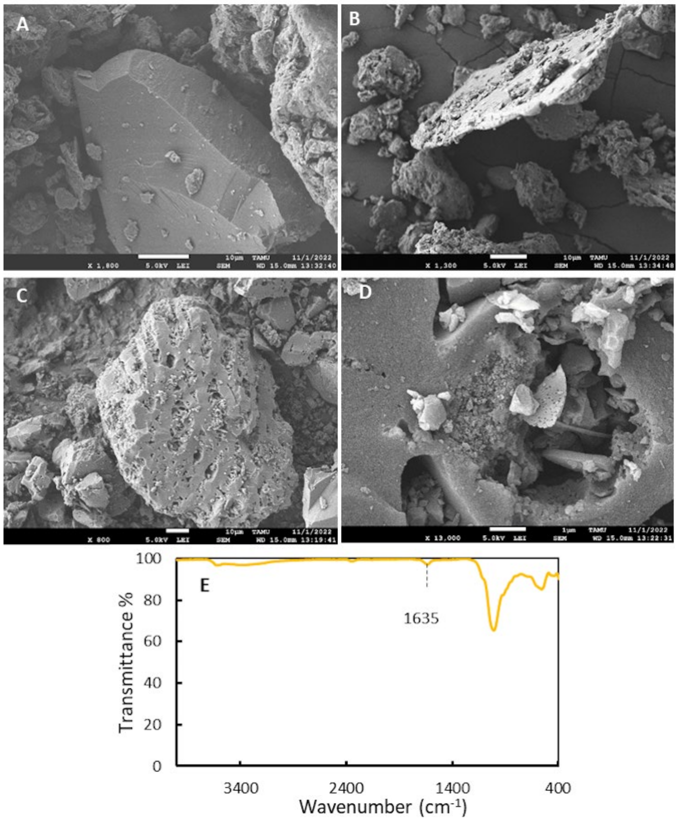

Figure S7: Scanning electron microscopy (SEM) images showing the flaky structure of CM-lactoglobulin (A, B) and the porous AC-lactoglobulin (C, D) with aggregates of lactoglobulin attached to the surfaces. FTIR spectra of CM-lactoglobulin (E).

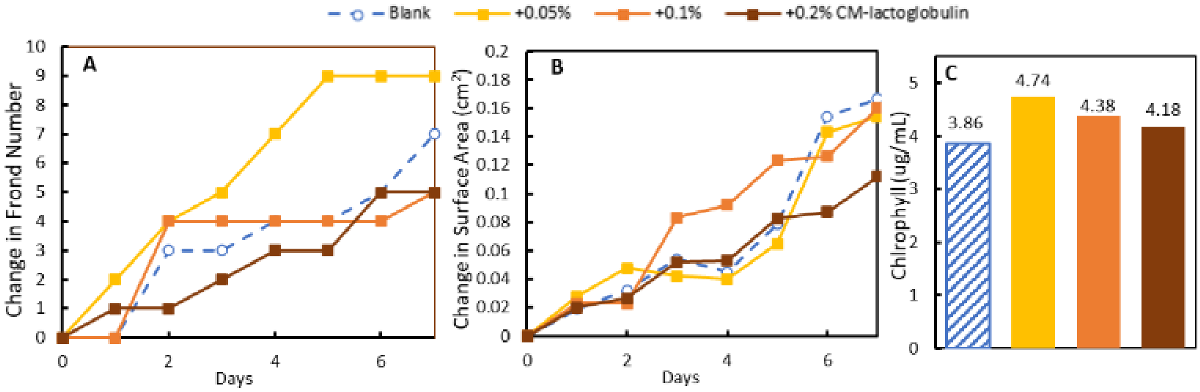

Figure S8: Effect of 0.05%, 0.1%, and 0.2% CM-lactoglobulin on changes of frond number (A), surface area (B), and chlorophyll content (C).

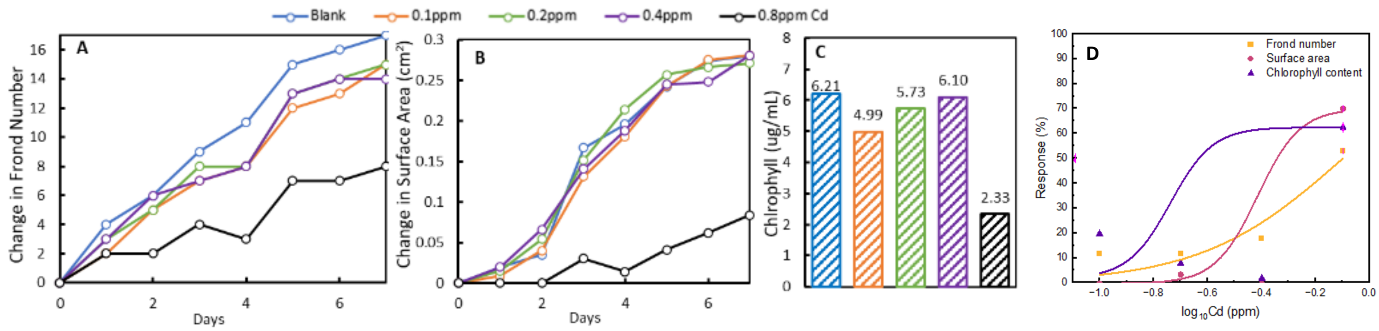

Figure S9: Dose-dependent toxicity of Cd at 0.1, 0.2, 0.4 and 0.8 ppm on changes of frond number (A), surface area (B), and chlorophyll content (C). Dose-response curve of Cd across parameters (D).

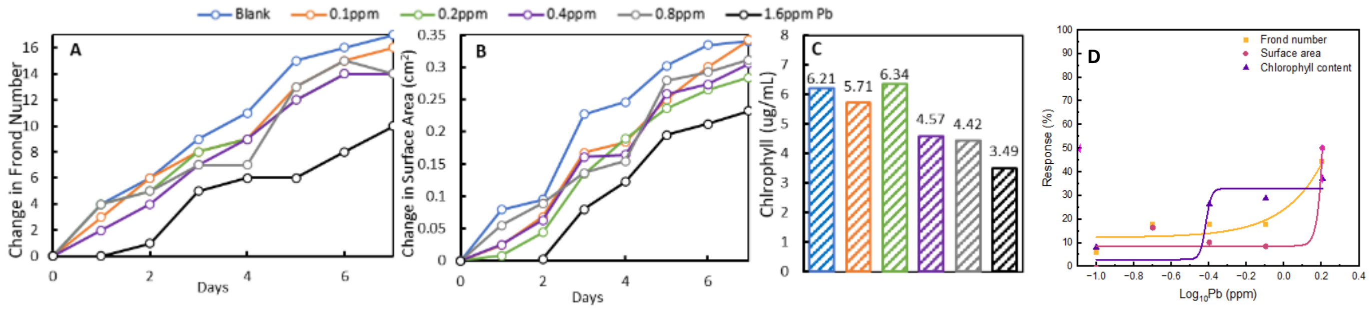

Figure S10: Dose-dependent toxicity of Pb at 0.1, 0.2, 0.4, 0.8, and 1.6 ppm on changes of frond number (A), surface area (B), and chlorophyll content (C). Dose-response curve of Pb across parameters (D).

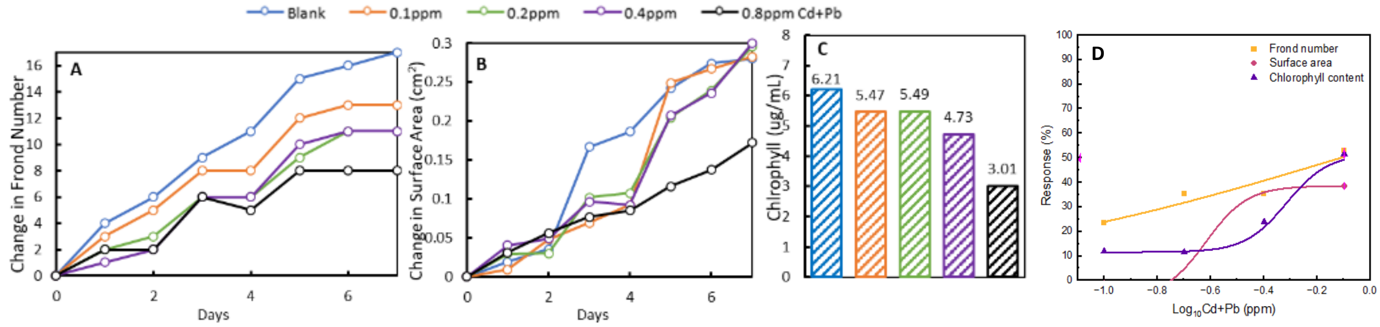

Figure S11: Dose-dependent toxicity of Cd and Pb mixtures at 0.1, 0.2, 0.4 and 0.8 ppm on changes of frond number (A), surface area (B), and chlorophyll content (C). Dose-response curve of Cd and Pb across parameters (D).
